# Magnetic Resonance Imaging and Molecular Dynamics Characterization of Ionic Liquid in Poly(ethylene oxide)-Based Polymer Electrolytes

**DOI:** 10.1021/acsami.0c01890

**Published:** 2020-04-30

**Authors:** Mosè Casalegno, Franca Castiglione, Guido Raos, Giovanni Battista Appetecchi, Stefano Passerini, Andrea Mele, Enzio Ragg

**Affiliations:** † Dipartimento di Chimica, Materiali e Ingegneria Chimica “G. Natta”, Politecnico di Milano, 20131 Milano, Italy; ‡ Snergy and Sustainable Economic Development, Materials and Physicochemical Processes Technical Unit, ENEA, Italian National Agency for New Technology, Via Anguillarese 301, 00196 Rome, Italy; § Helmholtz Institute of Ulm (HIU), Strasse 11, 89081 Ulm, Germany; ∥ Karlsruhe Institute of Technology (KIT), P.O. Box 3640, 76021 Karlsruhe, Germany; ⊥ Dipartimento di Scienze Molecolari Agroalimentari, Università di Milano, 20131 Milano, Italy

**Keywords:** Solid polymer electrolytes, magnetic resonance imaging, molecular dynamics simulations

## Abstract

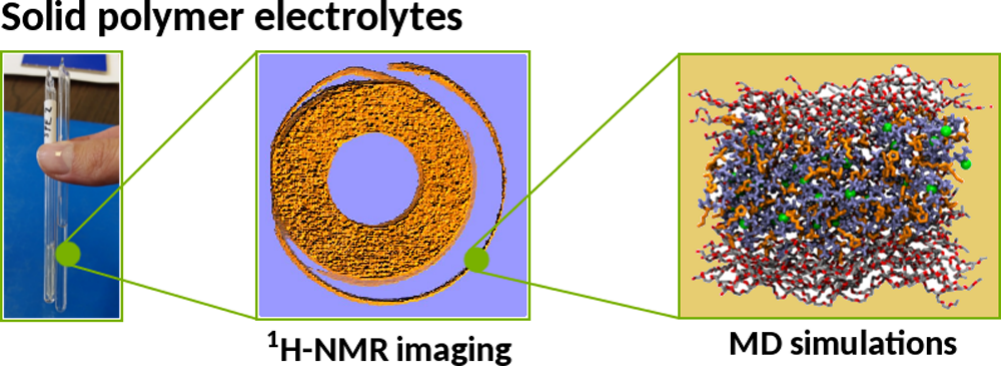

Ternary systems consisting of polymers, lithium salts, and ionic liquids (ILs) are promising materials for the development of next-generation lithium batteries. The ternary systems combine the advantages of polymer–salt and IL–salt systems, thus providing media with high ionic conductivity and solid-like mechanical properties. In this work, we apply nuclear magnetic resonance ^1^H microimaging [magnetic resonance imaging (MRI)] techniques and molecular dynamics (MD) simulations to study the translational and rotational dynamics of the *N*-butyl-*N*-methylpyrrolidinium (PYR_14_) cation in poly(ethylene oxide) (PEO) matrices containing the lithium bis(trifluoromethanesulfonyl) imide salt (LiTFSI) and the PYR_14_TFSI IL. The analysis of diffusion-weighted images in PEO/LiTFSI/PYR_14_TFSI samples with varying mole ratios (10:1:*x*, with *x* = 1, 2, 3, and 4) shows, in a wide range of temperatures, a spatially heterogeneous distribution of PYR_14_ diffusion coefficients. Their weight-averaged values increase with IL content but remain well below the values estimated for the neat IL. The analysis of *T*_2_ (spin–spin relaxation) parametric images shows that the PEO matrix significantly hinders PYR_14_ rotational freedom, which is only partially restored by increasing the IL content. The MD simulations, performed on IL-filled cavities within the PEO matrix, reveal that PYR_14_ diffusion is mainly affected by Li/TFSI coordination within the IL phase. In agreement with MRI experiments, increasing the IL content increases the PYR_14_ diffusion coefficients. Finally, the analysis of MD trajectories suggests that Li diffusion mostly develops within the IL phase, although a fraction of Li cations is strongly coordinated by PEO oxygen atoms.

## 1 Introduction

Solid polymer electrolytes (SPEs) represent the state-of-the-art for high safety, solvent-free media for lithium batteries^1−4^ and energy-storage applications.^5^ The traditional solid electrolytes are binary systems composed of a high-molecular-weight semicrystalline polymer, usually poly(ethylene oxide) (PEO) and its derivatives,^6−10^ and a lithium salt as the ion conductor.^11,12^ Battery performance is strongly influenced by the Li transport within the SPE matrix. This occurs through coordinated motions of chain segments and is primarily related to polymer properties such as their mobility, orientation, and degree of crystallinity, as Li transport occurs mainly within the amorphous phase. Unfortunately, because of the high degree of crystallinity of PEO, conventional SPEs offer very low ionic conductivity (in the range of 10^–8^ to 10^–5^ S cm^–1^ at room temperature), thus preventing their practical use. Among the solutions devised to improve SPE conductivities, the addition of molten salts such as ionic liquids (ILs)^13−18^ to form ternary mixtures was proven to be quite effective.

Although widely investigated by both experimental^15,19^ and computational^20−23^ methods, the effect of IL addition on the SPE structure and properties has only been partially understood. One important issue concerns the role of the IL in Li diffusion. So far, two hypotheses have emerged. According to the former,^20,21^ the IL acts as a PEO plasticizer and facilitates Li transport, which occurs predominantly within the polymer phase. Other studies,^12^ conversely, suggest that Li transport mostly develops in the IL phase, where lithium cations associate with the IL anions. Both mechanisms appear to play a role in real mixtures,^15^ their relative importance being significantly affected by the chemical and physical behavior of the IL within the host polymer.^19^ The quantification of the transport properties of the IL within these ternary blends is particularly important in this context and may help the development of a more comprehensive picture of ion transport in SPE systems.

So far, only a few studies have addressed IL diffusion in SPEs, with experimental data primarily provided by pulsed-gradient spin echo nuclear magnetic resonance (NMR).^19^ An alternative experimental approach involves the adoption of magnetic resonance imaging (MRI) techniques. In contrast to standard NMR, MRI is able to access spatially resolved information about distribution and transport of the probed molecular species and, therefore, is ideally suited for this kind of investigation. ^1^H MRI has been widely exploited in the medical area, where it has become a well-established noninvasive diagnostic tool to map water diffusion in brain^24,25^ and tissues.^26^ In the field of material science, MRI applications include characterization of heterogeneous materials,^27,28^ plastic crystal electrolytes,^29^ polycrystalline materials,^30^ and electrode microstructure in Li batteries.^31−33^ To our knowledge, the application of MRI to IL diffusion in SPE has not yet been attempted.

In this work, we apply two ^1^H MRI microimaging techniques, namely, diffusion MRI (dMRI) and spin–spin relaxation mapping (*T*_2_-MRI), to characterize the translational and rotational dynamics of the *N*-butyl-*N*-methylpyrrolidinium (PYR_14_) cation in PEO matrices containing the lithium bis(trifluoromethanesulfonyl) imide (LiTFSI) salt and the *N*-butyl-*N*-methylpyrrolidinium bis(trifluoromethanesulfonyl) imide (PYR_14_TFSI) IL. Our interest in these systems is motivated by their increasing importance in the field of Li batteries^34−37^ and the availability of experimental data.^15,19^ Our experimental findings are complemented by molecular dynamics (MD) simulations. These have been extensively used to model the IL transport as bulk liquids^38^ or in heterogeneous systems^20−23^ and will be adopted here to rationalize the experimental results and provide insights about ion dynamics in SPE systems.

## 2 Experimental Methods

### 2.1 Materials and Sample Preparation

**Figure 1:**
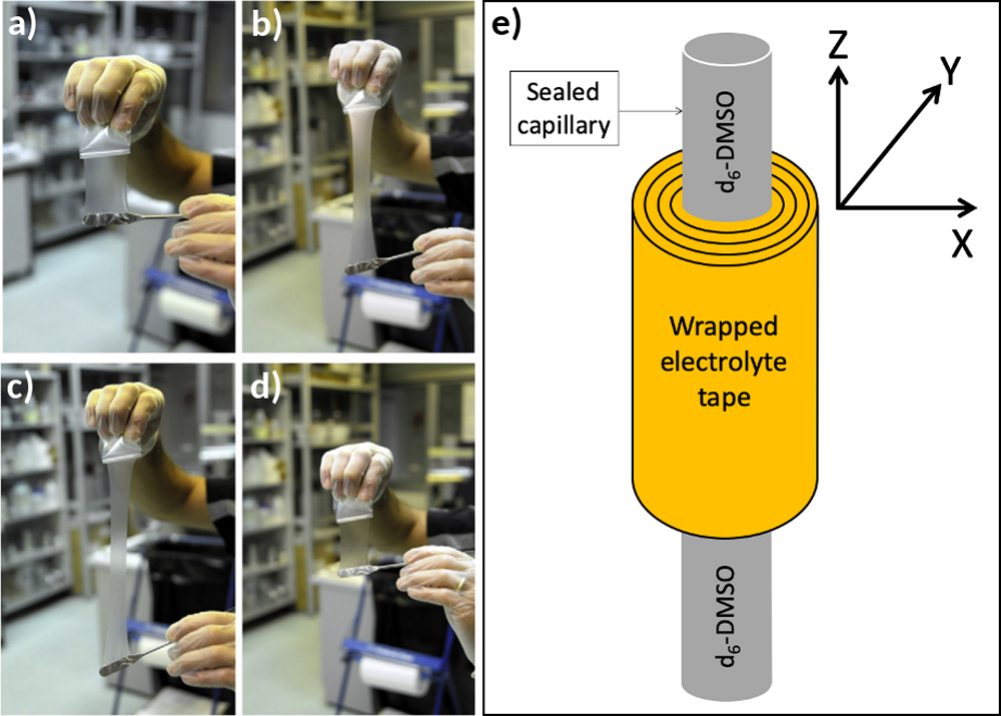
Panels (a–d): Pictures illustrating PEO/LiTFSI/PYR_14_TFSI film preparation. Panel (e): Scheme of the NMR sample. Instrument coordinates are also reported.

The PEO/LiTFSI/PYR_14_TFSI ternary polymer electrolytes were prepared through a solvent-free, hot-pressing procedure, previously reported,^13,15^ carried out in a dry room with a very low relative humidity (RH < 0.1% at 20 °C). The PYR_14_TFSI IL was synthesized and purified through a procedure described in detail elsewhere.^39^ LiTFSI (3 M, 99.9 wt %) and PYR_14_TFSI were vacuum dried at 120 °C for 24 h before use, whereas PEO (Dow Chemical, WSR 301, MW = 4 × 10^6^ g/mol) was dried under vacuum at 50 °C for 48 h. PEO and LiTFSI (with a EO/Li mole ratio equal to 10 and kept constant in all preparations)^14^ were intimately mixed in a mortar, and then PYR_14_TFSI was added. The LiTFSI/PYR_14_TFSI mole ratios were 1:1, 1:2, 1:3, and 1:4 for samples SPE2 to SPE5, in the order. A summary of the polymer composition is also present in Table 1. After complete blending, the PEO/LiTFSI/PYR_14_TFSI electrolyte paste was vacuum-annealed at 100 °C for 2–3 days to obtain a homogeneous, plasticlike material, which was hot-pressed (between two Mylar foils) at 70 °C and 180 kg cm^–2^ for 5 min for obtaining 0.1 mm thick films. The polymer electrolyte tapes, handled within the dry room, were carefully wrapped around a sealed capillary containing dimethyl sulfoxide (DMSO)-*d*_6_ (Figure 1a–d) and then housed in glass tubes, which were flame-sealed to avoid contamination from external sources (Figure 1e). Pure PYR_14_TFSI, housed within a flame-sealed glass tube containing DMSO-*d*_6_, was used as the reference (sample IL in Table 1). Additional pictures of the NMR samples are available in the Supporting Information (see Figure S1).

**Table 1:** Experimental Diffusion Coefficients *D*^exp^ and *T*_2_ Relaxation Times of PYR_14_ at 19 °C and at 50 °C^a^

sample	EO/Li/IL mole ratio	*D*^exp^ 19 °C [(10^–6^ mm^2^/s)]	*D*^exp^ 50 °C [(10^–6^ mm^2^/s)]	*T*_2_, 19 °C (ms)	*T*_2_, 50 °C (ms)
SPE2	10:1:1	0.9 ± 0.4	3.4 ± 1.4	21.0 ± 10	49 ± 15
SPE3	10:1:2	1.6 ± 0.4	6.2 ± 1.7	42.0 ± 10	51 ± 11
SPE4	10:1:3	2.1 ± 0.7	7.6 ± 1.5	58.0 ± 15	74 ± 31
SPE5	10:1:4	3.9 ± 0.5	12.5 ± 2.5	49.0 ± 12	110 ± 21
IL		16.2 ± 0.5	31.8 ± 3.9	91.9 ± 0.4	125 ± 1

a For better clarity, the sample composition has also been reported.

### 2.2 MRI Characterization

MRI studies were performed using a standard bore Bruker AVANCE II AV600 spectrometer (Bruker BioSpin GmbH, Rheinstetten, Germany) equipped with a 10 mm ^1^H microimaging probe and a variable temperature control unit. The magnetic field strength was 14 T, corresponding to the ^1^H resonance frequency of 600.1 MHz. For the acquisition of proton density (see Figure S3) and *T*_2_ parametric images, a series of MRI slices of SPE samples were preliminarily taken at temperatures between 15 and 50 °C, using a standard multislice multiecho pulse sequence with the following acquisition parameters: number of slices: 8; thickness: 0.3 mm; no slice separation; repetition time: 800 ms; echo time: 3.2 ms; number of echoes: 8; number of scans: 8; total acquisition time: 15 min. Field of view was set to 4.5 × 4.5 mm^2^, with a matrix size of 128 × 128 units, corresponding to an in-plane resolution of 35 × 35 μm^2^/pixel.

Diffusion measurements were performed using a diffusion tensor spin warp pulse sequence^40^ (Bruker DTI Standard pulse sequence). For each experimental condition (IL concentration and temperature), separate measurements were performed with the gradient direction along the *x*-, *y*-, and *z*-axes. A 2 mm slice thickness and an echo time (TE) of 20 ms (close to the minimum allowed value, TE = 18.5 ms) were chosen for better sensitivity (see Figure S2). Preliminary experiments showed that the derived values were independent of the thickness in the range between 0.5 and 2 mm. Other parameters relevant to the diffusion coefficient measurement are as follows: Δ = 300 ms (samples SPE3–SPE5); Δ = 400 ms (sample SPE2); δ = 5 ms; maximum *b*-value: 2,664,753 s/mm^2^; gradient scaling: 6.13% up to 51.25%; total number of gradients: 10; number of scans: 16; repetition time: 2.5 s; total acquisition time: 20 h. Because of restrictions in the pulse sequence timing, the field of view was increased to 8 × 8 mm^2^, with a matrix size of 128 × 128 pixels, corresponding to an in-plane digital resolution of 63 × 63 μm^2^/pixel. The vertical, out-of-plane resolution is 2 mm.

For each slice, *T*_2_ and *D* values were extracted by a multiparametric nonlinear fitting of the intensity decays (*y* = *a* + *b*e^–*t*/*T*_2_^ and *y* = *c* + *d*e^–*fD*^, respectively, with *a*, *b*, *c*, and *d* as parameters and *t* and *f* as vectors of given TEs and *b*-values) and used for reconstruction of the corresponding parametric images. Values are color coded from blue (low) to red (high), as reported in each figure. Field homogeneity was checked after careful shimming by acquiring a volume selective ^1^H NMR spectrum of SPE samples. The contribution of background gradients to the diffusion measurements was evaluated by varying Δ in the 150–400 ms range. A negligible variation, well below the reported standard deviations, was observed for all measurements (deviations were 5 and 8% for Δ = 150–300 and Δ = 150–400 ms, respectively). Results are reported in the Supporting Information (see Figures S8–S11).

Acquisition, data processing, and image analysis were performed with ParaVision v.4.0 (Bruker BioSpin MRI GmbH, Ettlingen, Germany).

### 2.3 MD Simulations

**Figure 2:**
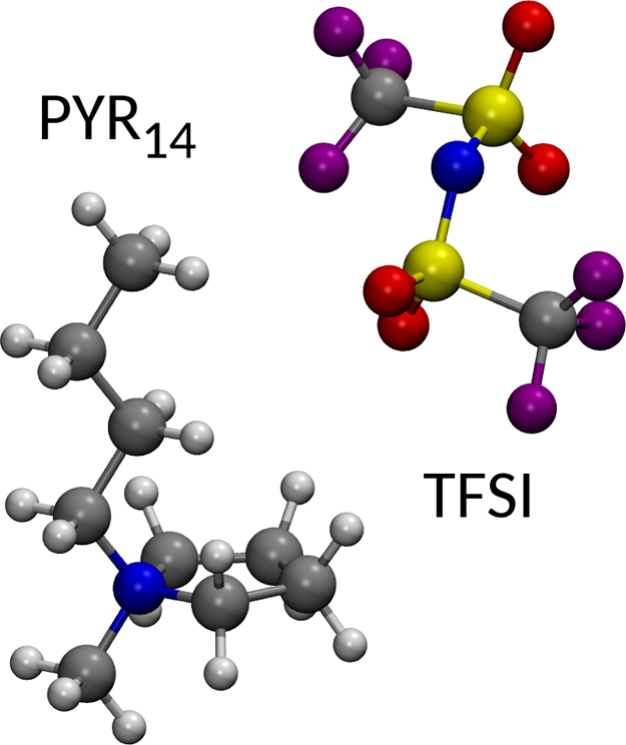
Sketches of the molecular structures of the PYR_14_ cation and the TFSI anion.

To characterize interactions and dynamics within the three-component system, simulation boxes were set up to model IL-filled cavities within PEO. Periodic boundary conditions were applied along all axes. The boxes were initially filled with 144 isodirectional PEO chains, aligned along the *z*-axis, consisting of 30 monomers each. Each chain, initially fully stretched, was made infinitely periodic along the main chain axis, so as to better account for realistic polymer lengths. In practice, this was obtained by linking each chain to its periodic image across the simulation box. During the subsequent step, 74 PEO chains were manually removed from the box in order to create a cylindrical empty hole across the box, parallel to the *z*-axis. The number of PEO chains after the removal was 70. The cylindrical hole was filled with LiTFSI/PYR_14_TFSI mixtures with different Li/PYR_14_ mole ratios. A sketch of the molecular structures of PYR_14_ and TFSI molecules is given in Figure 2.

The ratios were chosen to match those of SPE2–SPE5 samples (Table 1). The overall number of TFSI anions was constant in each system and set to 120. The number of Li ions was set at 60, 40, 30, and 24. Accordingly, the number of PYR_14_ ions was set at 60, 80, 90, and 96. For comparison purposes, one additional system not containing Li was also considered and will be hereafter denoted as SPE*N*. Note that the PEO concentration in the simulated systems was lower than that in the real samples. This choice was dictated by the need to limit the system size, keeping the computational cost of the MD simulations at a manageable level.

All MD simulations were performed at a constant temperature and pressure (1 atm) with the package Gromacs 5.0.7.^41^ The intra and intermolecular interactions of PYR_14_ and TFSI were modelled by means of the Pádua–Canongia Lopes force field,^42,43^ whereas OPLS-all atom parameters^44^ were used to model the remaining molecular species (Li cation and PEO). The cutoff radius for nonbonded interactions was set at 1.2 nm. The equations of motion were propagated with a leap frog algorithm with a time step of 1 fs. The system temperature was kept constant via velocity rescaling,^45^ with a time constant of 0.5 ps. The pressure was controlled via isotropic coupling to a Berendsen barostat,^46^ with a time constant of 1.0 ps and isothermal compressibility of 1 × 10^–5^ atm^–1^. Electrostatic interactions were treated via the particle-mesh Ewald method^47^ with a Fourier grid spacing of 0.12 nm. During the preliminary stage of our work, all systems were equilibrated at 30 °C for 50 ns. After equilibration, the final box size was approximately 4.7 × 5.8 × 6.4 nm^3^. Production runs were performed within the same *NPT* ensemble at 19 °C for 500 ns.

## 3 Results and Discussion

### 3.1 Diffusion Imaging (dMRI)

The basic diffusion sensitive MRI experiment consists of applying a train of pulsed field gradients (PFGs) of duration δ and increasing intensity along a defined axis (*z*, conventionally). The PFGs are separated by a delay Δ, which defines the timescale of the diffusion measurement. In simple isotropic systems, where the motion of the diffusive species obeys Fick’s laws, the signal decay intensity can be related to the mean square displacement (MSD) as
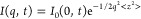
1where ⟨*z*^2^⟩ is the MSD along the gradient direction. In the case of the so-called Fickian diffusion, this scales linearly with time
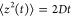
2

Equation 2 is commonly used in NMR practice to obtain a single value of the diffusion coefficient via log–log regression analysis.

In addition to *D*, the diffusive motion may also be characterized by *G*(*z*,*t*), the probability that an ion undergoes displacement *z* over time *t*. Given the latter, one may always compute the MSD
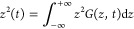
3

The opposite operation (i.e., obtain *G*(*z*,*t*) from the MSD) is not always possible. In this sense, *G*(*z*,*t*) is much richer in information. One important but special counter example is that of a simple, homogeneous, and isotropic system, as the distribution can then be expected to follow the Gaussian behavior
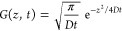
4

Significant deviations from the Gaussian behavior may be expected when the ions diffuse in a spatially heterogeneous medium, leading to the observation of coexisting populations of fast- and slow-diffusing species (the origin of the heterogeneity may not be always obvious, though). This is conveniently expressed by introducing a distribution of diffusion coefficients, *P*(*D*), from which the average diffusion coefficient can be estimated as
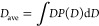
5

One advantage of MRI over standard NMR techniques is the possibility to spatially resolve the distribution of diffusion coefficients, thereby giving *P*(*D*).

**Figure 3:**
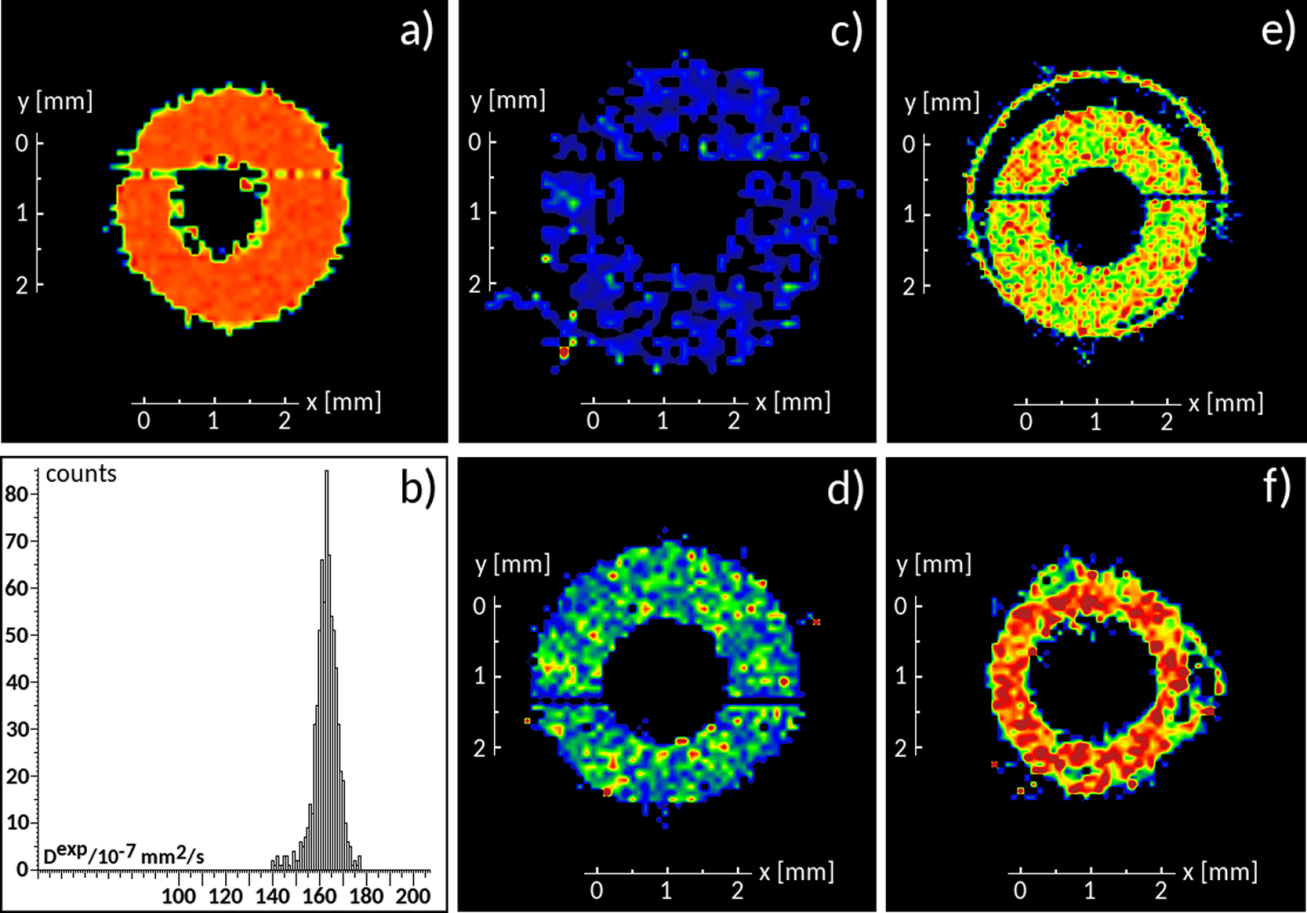
(a) DW image of neat PYR_14_TFSI at 19 °C; (b) corresponding diffusivity histogram. DW images at 19 °C for the samples (c) SPE2, (d) SPE3, (e) SPE4, and (f) SPE5. For better clarity, the diffusion coefficients are color-coded as follows: (a) 256 gradations from blue (*D* = 1 × 10^–6^ mm^2^/s) to red (*D* = 200 × 10^–6^ mm^2^/s); (c–f): 256 color gradations from blue (*D* = 0.1 × 10^–6^ mm^2^/s) to red (*D* = 15 × 10^–6^ mm^2^/s). In all DW images, the *xy* resolution is 63 × 63 μm^2^/pixel.

Figure 3a shows a proton diffusion-weighted (DW) image of PYR_14_ in a sample of neat PYR_14_TFSI at 19 °C. The view is along the *z*-axis, that is, the direction of the applied magnetic field. As expected for an isotropic and homogeneous liquid, there is no significant color gradient. The distribution of diffusivities (panel b) is narrow, with 0.5 × 10^–6^ mm^2^/s standard deviation, and centered at 16.2 × 10^–6^ mm^2^/s, representing the average diffusion coefficient of this species as already reported in the literature.^15^

The remaining panels in Figure 3 (namely, c, d, e, and f) show the DW images of a PEO/LiTSFI/PYR_14_TFSI mixture with increasing PYR_14_TFSI content, as specified in Table 1. The high-resolution microimages show the translational dynamics of the PYR_14_ cation in the SPE materials, with an in-plane resolution of a few micrometers. As in the previous case, the samples are oriented along the instrumental *z*-axis (see Figure 1e). The DW signal in a pixel results from the total contribution of all PYR_14_ ions within a voxel, whose dimensions are 63 μm × 63 μm × 2 mm. We note that these values cannot be associated with the internal motion of PEO hydrogens, which is expected to occur at timescales far beyond those accessible to MRI (milliseconds).

**Figure 4:**
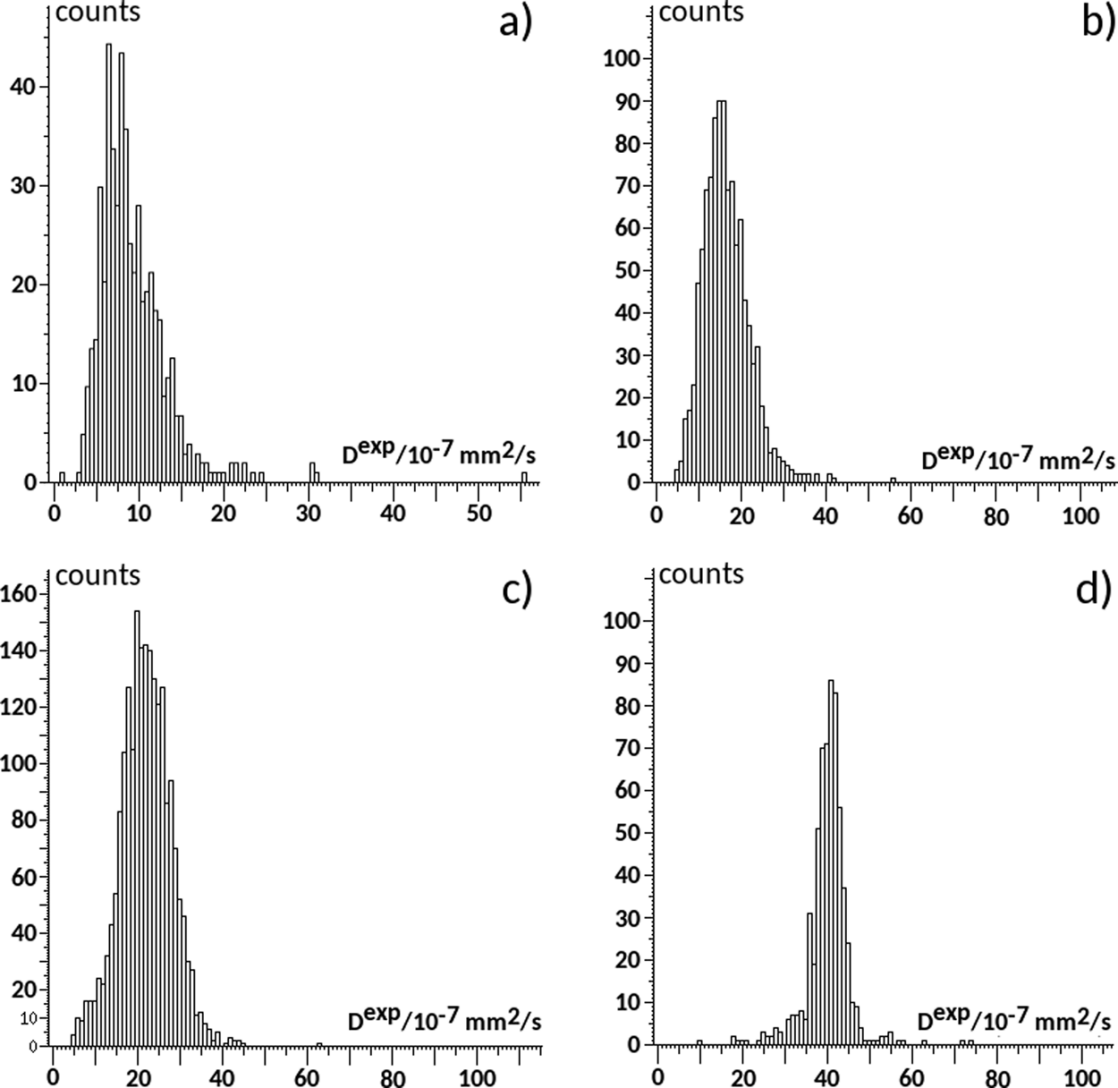
Diffusivity histograms at 19 °C for the samples (a) SPE2, (b) SPE3, (c) SPE4, and (d) SPE5.

In contrast to the neat IL, the images clearly indicate that cation dynamics is less homogeneous. Some noise is visible in SPE2 because of the low IL content. The heterogeneous character of PYR_14_ diffusion appears evident in Figure 4, where the corresponding diffusivity histograms are reported. All distributions are broadly centered on their averages. In all samples, small, asymmetric, right-extending tails suggest a small fraction of cations with higher diffusion coefficients. Table 1 collects the average PYR_14_ diffusion coefficients, calculated according to eq 5 at 19 °C and at 50 °C.

Increasing the IL content was found to enhance PYR_14_ diffusion, although far below the values observed in neat IL samples, both at 19 and 50 °C. Attempts at increasing the IL mole ratio beyond 4, as in the SPE5 sample, failed because of IL leakage.^19^ As we shall see in the following paragraph, where we describe *T*_2_ measurements to get further information about molecular mobility, SPE5 composition might correspond to a saturated sample condition, where IL starts to separate from PEO, giving rise to less homogenous samples.

### 3.2 *T*_2_ Imaging (*T*_2_-MRI)

The *T*_2_ relaxation time defines the time constant for the decay of transverse magnetization, commonly referred to as spin–spin relaxation^48^
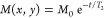
6

It is the result of random dynamic processes due to spin–spin interactions. According to the Bloembergen–Purcell–Pound (BPP) theory for pure liquids,^49^ the spin–spin relaxation rate, defined as 1/*T*_2_, is related to the molecular rotational correlation time τ_c_
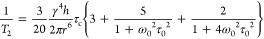
7where γ is the nuclear gyromagnetic ratio of the observed nucleus, *h* is the Planck constant, *r* is the distance between the interacting spins, and ω_0_ is the Larmor frequency at the magnetic field used for the measurement. From a practical point of view, it is suffice to say that small molecules freely tumbling in isotropic, nonviscous solvents typically show long *T*_2_ values, whereas macromolecules or small molecules with constrained motion are characterized by short *T*_2_ values. In addition to these spin–spin interactions, the transverse magnetization is also perturbed by small local magnetic field inhomogeneity Δ*B*_0_ because of the sample morphology. The corresponding effective relaxation time is called *T*_2_^*^ and obeys the equation
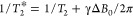
8

Clearly, 1/*T*_2_^*^ > 1/*T*_2_ because Δ*B*_0_ > 0 (it represents the root-mean-square deviation in the magnetic field strength) and γ > 0 for ^1^H, so that the BPP theory provides a lower bound for the rate of decay of the transverse magnetization in heterogeneous systems.

**Figure 5:**
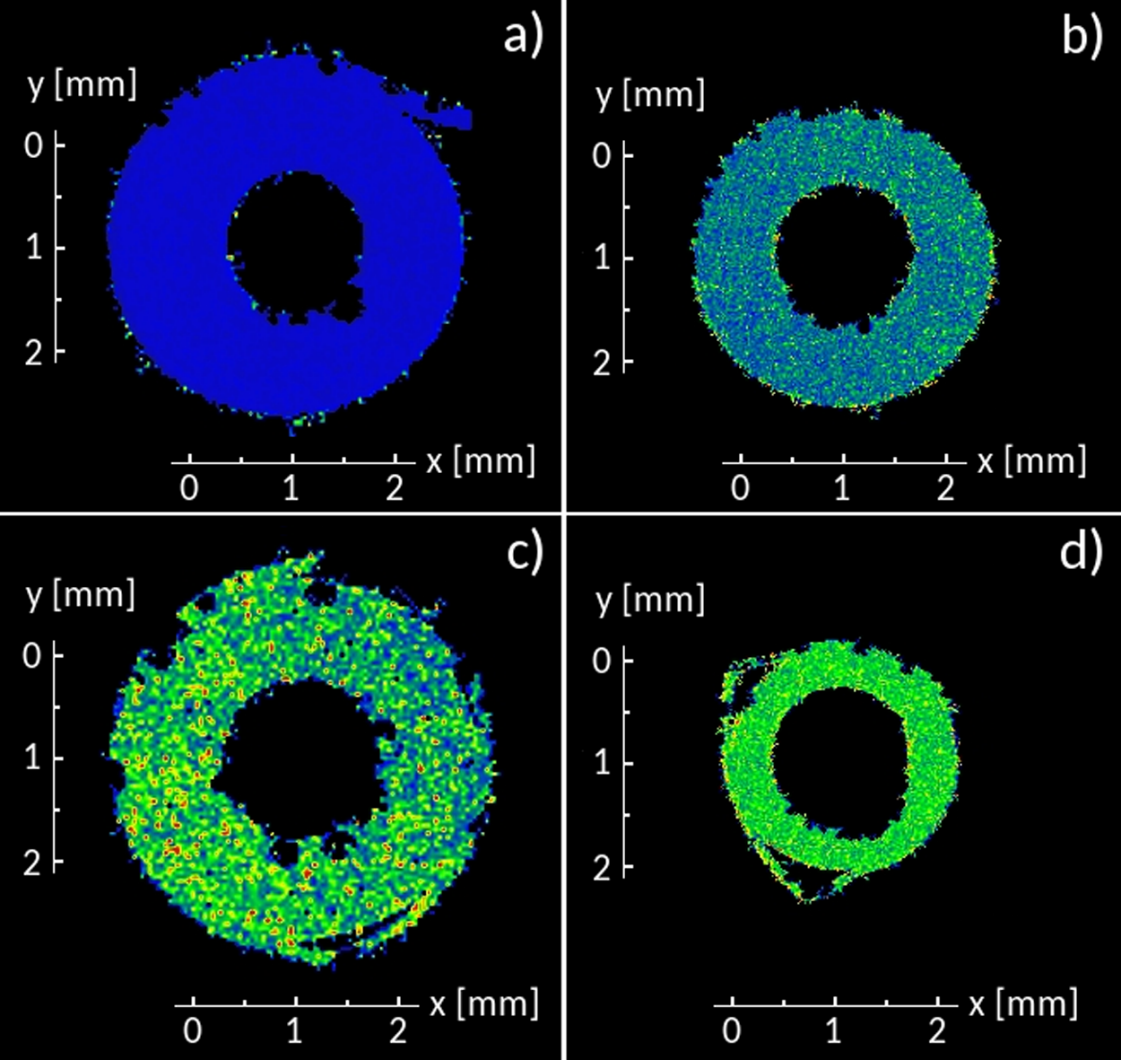
*T*_2_-weighted MRI images for the samples: (a) SPE2, (b) SPE3, (c) SPE4, and (d) SPE5 at 19 °C. *T*_2_ values are color-coded as 256 color gradations from blue (*T*_2_ = 9 ms) to red (*T*_2_ = 90 ms).

The proton *T*_2_-weighted MRI images for the four samples SPE2–SPE5 are reported in Figure 5. The color scale from blue to red indicates an increasing *T*_2_ value because of an increasing tumbling rate (decreasing rotational correlation time τ_c_) of the IL in the polymer matrix. The different color shades, from blue for SPE2 to green-yellow for SPE5, suggest that the mobility of the PYR_14_ cation increases with the amount of IL. In particular, for sample SPE2 (panel a in Figure 5), a more homogeneous distribution of relaxation times is observed. Consequently, the histogram of the *T*_2_ values (see Figure S4) has a single, sharp peak. Increasing the amount of IL, as for samples SPE3 (panel b) and SPE4 (panel c), a green-blue colored map is observed indicating regions characterized by an inhomogeneous rotational motion. The neat IL sample, taken as the reference, has greater mobility and a homogeneous *T*_2_ map, as indicated by the largely lower standard deviation.

Table 1 collects the spin–spin relaxation times *T*_2_ for all studied samples at two different temperatures (19 and 50 °C). The experimental values are in the range 32–50 ms (at 19 °C) for the SPE samples, compared with 91.9 ms observed for the neat IL. Increasing the temperature to 50 °C, the measured *T*_2_ values increase for all samples, consistently with a reduction in the rotational correlation time, τ_c_. In all cases, bulklike IL behavior is ruled out. The short transverse relaxation *T*_2_ values indicate that the polymer matrix hinders the tumbling of PYR_14_ cations in all SPE samples. This IL–polymer interaction may contribute to the plasticizing effect of IL on PEO/LiTFSI systems and to the inhibition of crystallization promoted by the added IL. As reported in ref (14), the glass transition for PEO/LiTFSI (10:1) decreased from −39 to −55 °C after addition of 1 equiv of PYR_14_TFSI (ternary system PEO/LiTFSI/PYR_14_TFSI 10:1:1, i.e., SPE2).

### 3.3 MD Simulations

**Figure 6:**
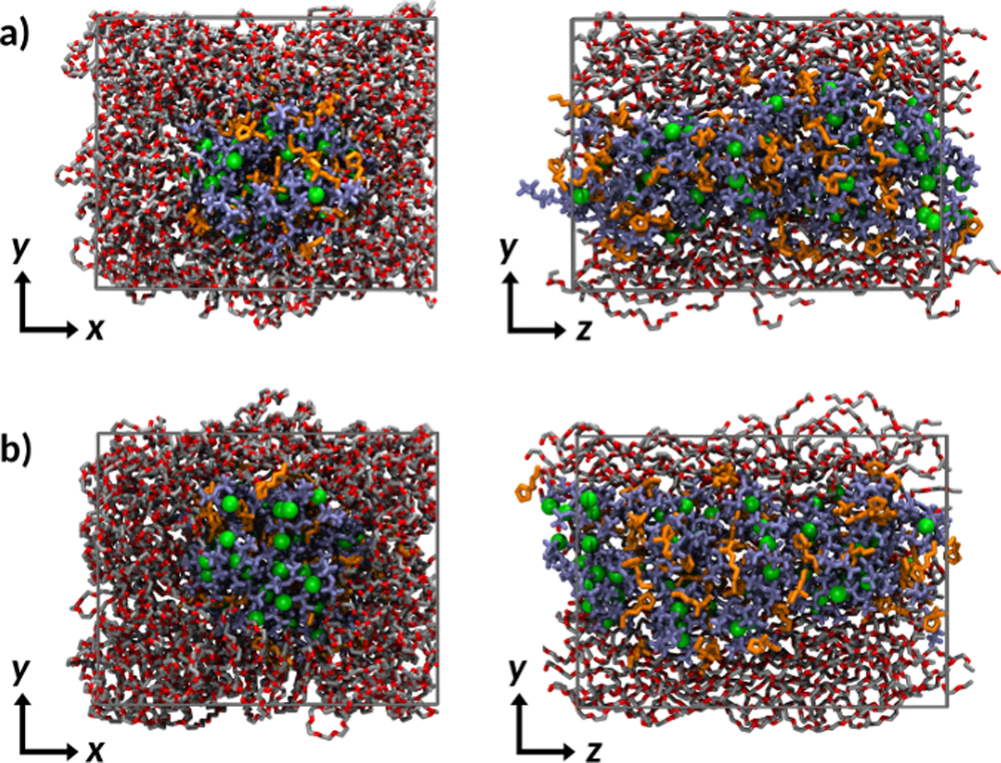
Front (along *z*-axis) and side (along *x*-axis) views of the simulation box for the SPE2 system. PEO chains (red/gray) surround the PYR_14_ (orange)/TFSI (purple) IL. Li cations are highlighted in green. (a) Starting structure (after equilibration); (b) final structure (*t* = 500 ns).

Prompted by the MRI findings, we performed fully atomistic MD simulations on systems closely resembling the experimental samples with respect to the LiTFSI/PYR_14_TFSI ratio (see the Experimental Methods and Table 2 for details). For simplicity, we shall adopt the same nomenclature and indicate them by SPE2–SPE5. Figure 6 shows two views of the equilibrated SPE4 system. In contrast to other computational studies in the literature,^20−22^ in which IL and PEO form a single phase, in our model, these two components segregate to form distinct nanosized phases. This choice was guided by our interest in modelling the structure and ion dynamics at the interface between PEO and the IL. On the other hand, the same morphologies are retained also after 500 ns of MD for each system. Such simulation times correspond to the current state-of-the-art for IL simulations and are comparable or longer than those explored in the previous MD studies of PEO/IL mixtures.^20−22^

**Table 2:** Calculated (*D*^sim^) Diffusion Coefficients at 19 °C for Hydrogen Atoms of PYR_14_, Calculated by Integration of the *P*(*D*) Profiles at *t* = 400 ns [Eq 4]^a^

sample	EO/Li/IL mole ratio	*D*^sim^ [10^–6^ mm^2^/s]	*D*^exp^ [10^–6^ mm^2^/s]
SPE2	210:60:60	0.610	0.9 ± 0.4
SPE3	210:40:80	0.563	1.6 ± 0.4
SPE4	210:30:90	0.768	2.1 ± 0.7
SPE5	210:24:96	0.868	3.9 ± 0.5
SPE*N*	210:0:120	0.123	
IL		0.258	16.2 ± 0.5

a For comparison purposes, the experimental diffusion coefficients at 19 °C have also been reported (see Table 1).

As a first step in our analysis, we considered the translational diffusion of the PYR_14_ cations, as these are more directly comparable with the dMRI experiments. To this end, the spatial displacements of their hydrogen atoms were extracted from the MD simulations and averaged at different correlation times t, yielding the van Hove self-correlation function^50^
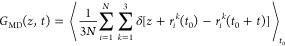
9

Here, the outer summation runs overall all hydrogen atoms (*i* = 1, ..., *N*) and the inner one over all spatial directions (*k* = 1, 2, 3), whereas the angular brackets indicate an average over all possible time origins *t*_0_. The average over all spatial directions cancels the possible effects of anisotropic diffusion along and orthogonal to the main axis of the cell in Figure 6, allowing a more meaningful comparison with the experiments.

**Figure 7:**
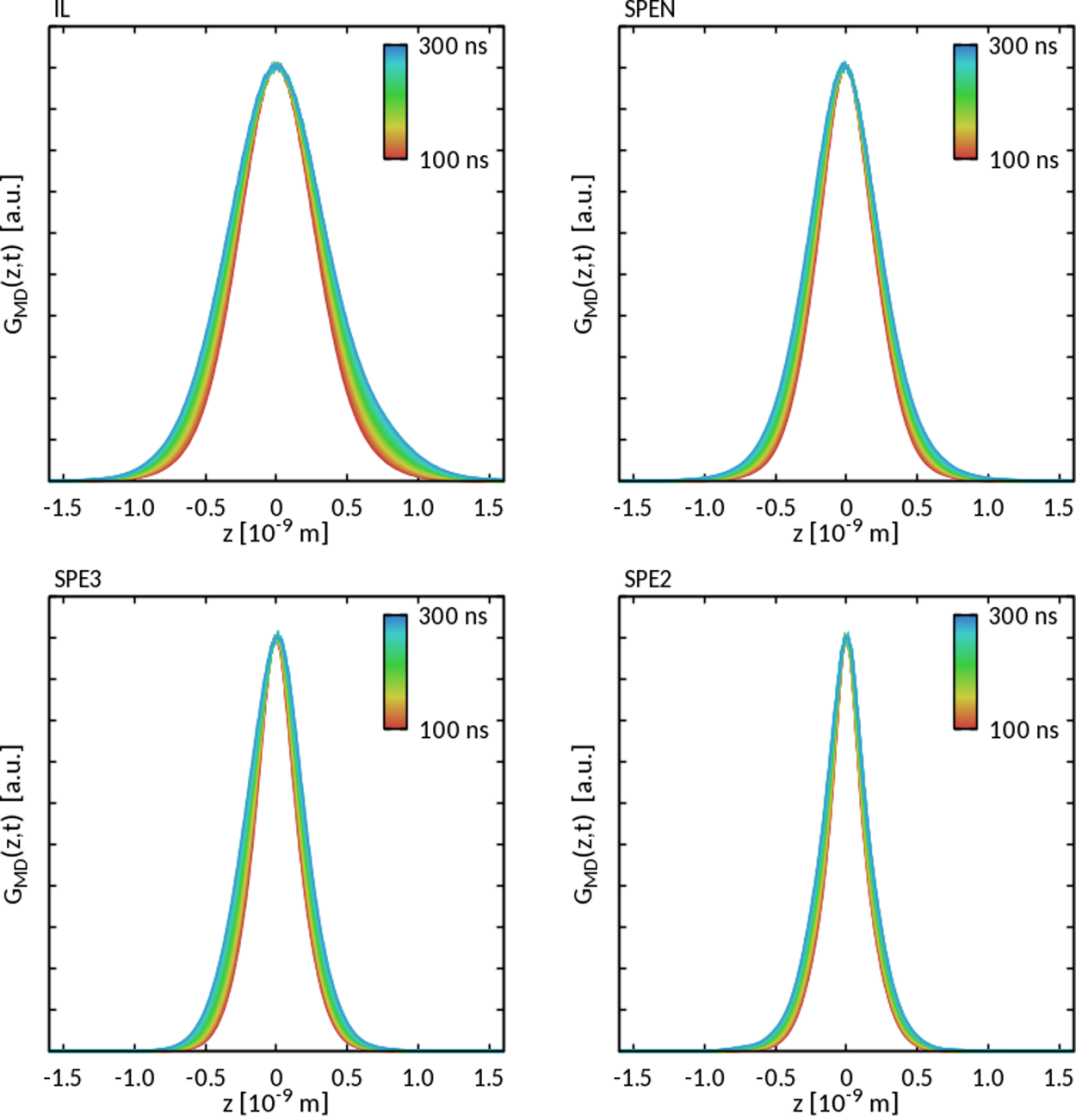
Hydrogen displacement distribution profiles for IL, SPE*N*, SPE3, and SPE2 in the range between 100 and 300 ns.

Figure 7 shows the displacement profiles associated with PYR_14_ diffusion for the neat IL and different PEO-containing systems. As expected, the displacement distributions widen over time. Consistently with dMRI results, the width of the displacement distributions, for any given value of *t*, decreases on going from the neat IL to SPE2, for example, with decreasing IL content.

In order to compare these results with dMRI and investigate the possibility of multiple transport regimes, we extracted the distribution of diffusivities from the displacement distribution profiles by Lucy’s deconvolution algorithm.^51^ In doing so, we assumed that *G*_MD_ could be described as the superposition of elementary Gaussian processes, for example
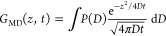
10where *P*(*D*), the probability distribution of diffusivities, is analogous to that introduced above (see eq 5). The fitting of *G*_MD_ was performed at different correlation times. The resulting diffusion coefficients evidenced a converging behavior upon increasing the correlation time (see Figure S5). Below, we present the results at *t* = 400 ns. This choice represents a good compromise between the conflicting requirements of describing long-time diffusive motions and retaining good statistical accuracy because the latter degrades when *t* approaches the total MD simulation time (500 ns) because of the smaller number of data points.

**Figure 8:**
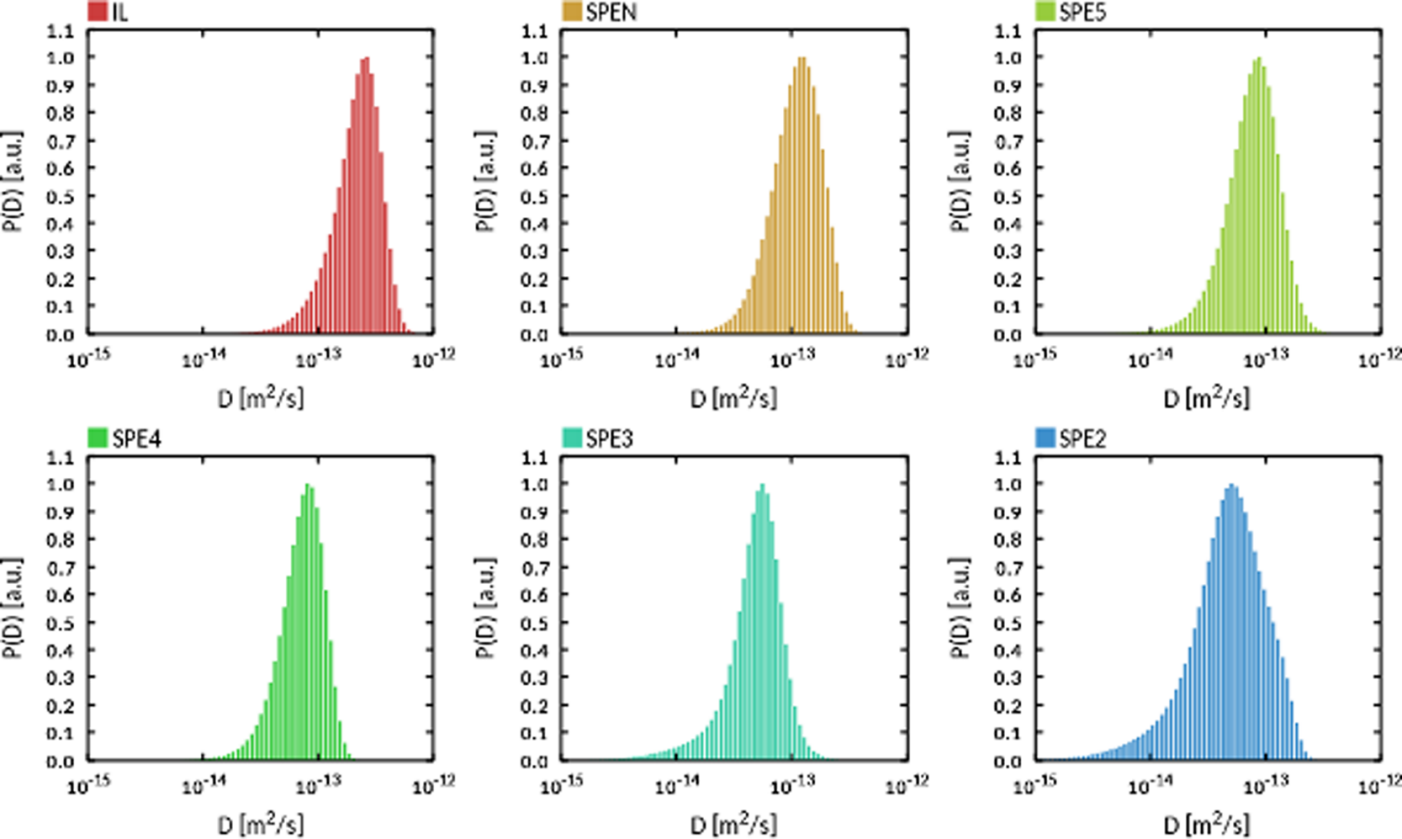
*P*(*D*) profiles for hydrogens obtained by fitting the corresponding displacement distributions at 19 °C for *t* = 400 ns.

The results of the fitting procedure are reported in Figure 8. All *P*(*D*) profiles are single peaked and slightly asymmetric. This type of distribution rules out the possibility of distinct transport regimes at timescales approaching microseconds, consistently with the dMRI results in the millisecond timescale. The result obtained for the IL is analogous to that obtained in our previous work on the same IL^38^ and consistent with a molecular picture where the ions move in transient cages formed by their nearest neighbors. Large spatial displacements, triggered by occasional cage rearrangements, are responsible for the right-extending tails observed in *P*(*D*). These caging effects can be expected to disappear for the IL in the limit of long time scales, when ordinary diffusion eventually takes over.

The *P*(*D*) profiles obtained for the remaining systems are similar to those of IL. Decreasing the PYR_14_ fraction (e.g., increasing the Li content) shifts the maximum of the distribution toward lower values, consistently with the experimental results presented above (see Figure 4). At the same time, increasing the correlation time narrows the *P*(*D*) profiles, suggesting that, in the long time limit, the fraction of fast diffusing molecules would be much inferior to the experimentally observed one (see Figure 4). We believe that this difference can be ascribed to the limited ability of model systems to account for the number of structurally diverse environments that characterize a real macroscopic sample.

To estimate the average diffusion coefficients of PYR_14_, the diffusivity profiles were integrated using eq 5, the same equation adopted to obtain experimental DW averages. Table 2 collects the average diffusivities for all systems investigated.

Overall, the calculated and experimental estimates of the PYR_14_ diffusion coefficients (Tables 2 and 1, respectively) follow the same trends with respect to composition. We shall show below that our results for Li diffusion are consistent with the experimental estimates reported in the literature. The hydrogen diffusion coefficient in the neat IL is about twice that in the SPE*N* sample, which does not contain Li, suggesting that the PEO matrix alone may hinder PYR_14_ diffusion. Decreasing the IL fraction decreases the average diffusion coefficients of PYR_14_. However, there are also important quantitative differences between the simulations and experiment. This can be traced to the approximations and assumptions underlying our MD simulations. These include the choice of the initial morphologies and the limited system sizes and simulation times. As noted elsewhere,^22,52,53^ the choice of the force field may have a significant impact on the simulation results. In particular, accurate results for the diffusion coefficients in ILs can only be expected after accounting for ion polarization effects,^54^ although the common practice of charge rescaling has also shown to provide valuable results.^53,55,56^ Nonpolarizable force fields typically underestimate diffusion coefficients by at least 1 order of magnitude, in line with our present results.^57,58^

**Figure 9:**
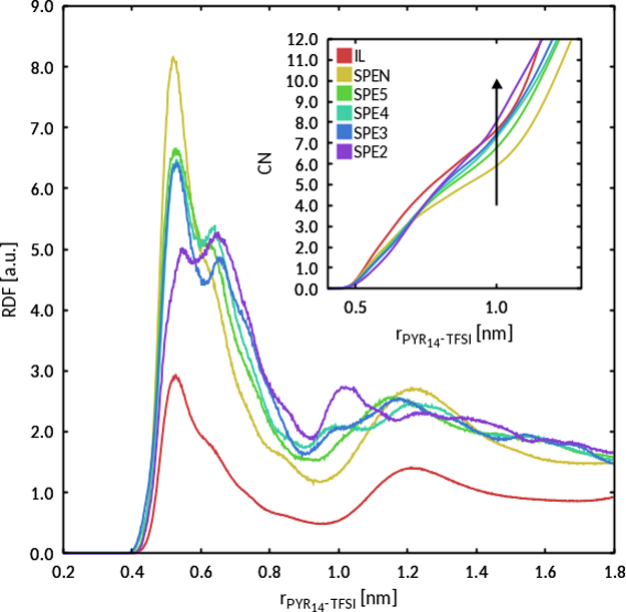
Center-of-mass, normalized pairwise RDF of PYR_14_/TFSI obtained from MD simulations at 19 °C. The corresponding CNs (the average number of TFSI anions within a given distance from a PYR_14_ cation) are shown in the inset.

In order to rationalize the observed trend in PYR_14_ diffusion coefficients, the MD trajectories were processed to extract the pairwise radial distribution functions (RDFs) between system constituents. These functions have been widely used to study the structure of ILs.^59,60^Figure 9 collects the RDFs for PYR_14_/TFSI, calculated with respect to the ions’ center-of-mass. The RDFs of IL and SPE*N* are closely similar, indicating equivalent solvation patterns of PYR_14_ in TFSI. The RDFs exhibit similar, well defined, solvation shells. The spatial extent of the former is defined by the position of the first minimum at about 1.0 nm. The coordination numbers (CNs) show a significant overlap, indicating that the presence of the polymer does not affect PYR_14_/TFSI coordination. This observation supports the idea of a weak interaction between IL and PEO, consistently with experimental evidence about the instability of binary PEO–IL mixtures.^15^ The PYR_14_/TFSI RDFs of the SPE systems appear less well defined than those of Li-free ones. Going from SPE5 to SPE2, a second maximum appears, not occurring in Li-free systems. In all systems, the first minimum occurs at about 1.0 nm, although with increasingly large fluctuations upon approaching SPE2. The successive coordination shells are not well defined, indicating that the presence of Li significantly alters PYR_14_/TFSI coordination. This is further confirmed by the CNs, which indicate that the average number of TFSI anions surrounding PYR_14_ cations is, at a given distance, higher for Li-containing systems.

**Figure 10:**
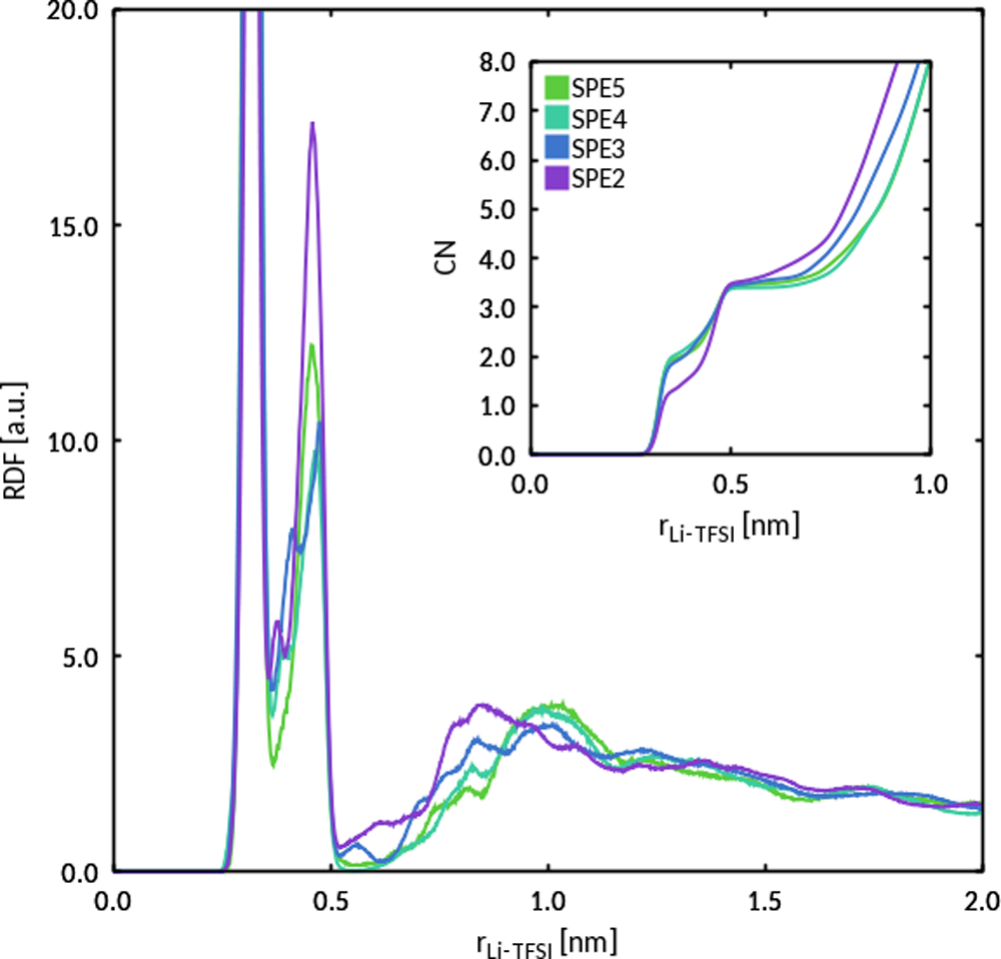
Center-of-mass, normalized pairwise RDF of Li/TFSI obtained from MD simulations at 19 °C. The corresponding CNs (average number of TFSI anions within a given distance from a Li cation) are shown in the inset.

**Figure 11:**
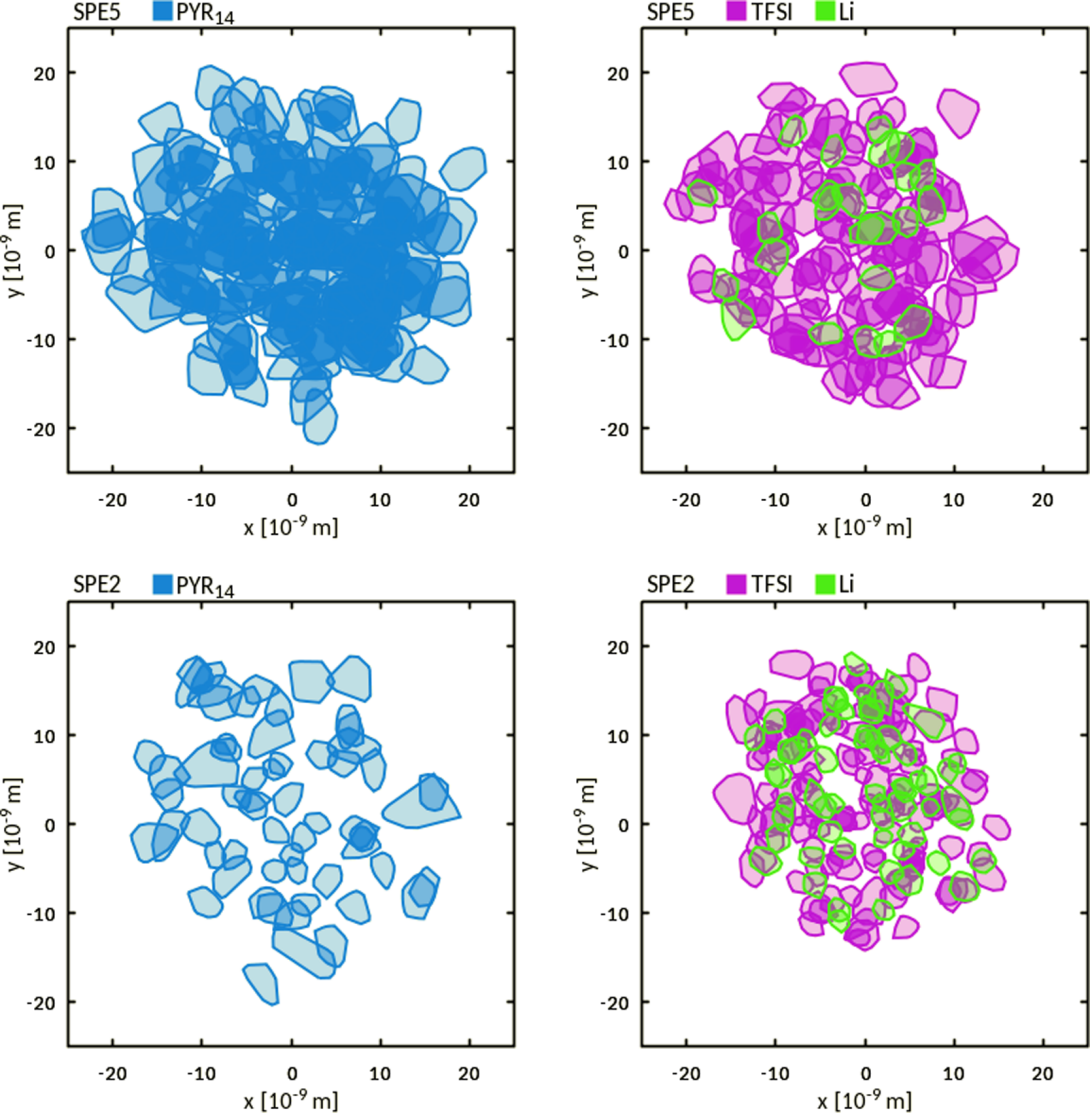
2D (over the *xy* plane) convex hulls of molecular center-of-mass coordinates sampled by PYR_14_ (left panels), TFSI, and Li (right panels) during the MD simulations for SPE5 (top) and SPE2 (bottom). For clarity, the hulls’ vertices were shifted and centered at the origin of the coordinate system.

To further investigate this outcome, we calculated the Li/TFSI RDFs for our systems. The result, shown in Figure 10, indicates that Li cations closely coordinate to TFSI anions. The RDFs are characterized by two minima at 0.35 and 0.51 nm, corresponding to two distinct coordination shells. The coordination numbers of 1.8 and 3.2 were obtained by integrating the RDFs up to these minima for all systems, except SPE2, for which the integration gave CN = 1.2 for the first minimum. The short-range association between Li and TFSI suggests that the attractive interaction between these species may hinder PYR_14_ diffusion, being therefore the main factor for the observed trend in PYR_14_ diffusion coefficients. In order to test this hypothesis, we analyzed the dynamics of PYR_14_, TFSI, and Li by extracting the spatial displacements (relative to the center-of-mass) from the full (500 ns) MD trajectories. To quantify the boundaries of these regions, we calculated for each ion the corresponding convex hull two-dimensional (2D) surface. The convex hull is defined as the smallest convex polygon surrounding a set of points in Euclidean space and is used here to quantify the maximum extension of the space sampled by each ion during the simulation. Figure 11 shows the 2D convex hulls for PYR_14_, TFSI, and Li for the SPE2 and SPE5 systems. The calculation was performed on molecular centers of mass. For simplicity, the hulls were calculated on the *xy* plane, irrespective of the molecular positions and displacements along the *z*-axis.

As already observed in other IL-containing systems,^38,61−63^ the existence of spatially limited regions results from the formation of transient cages where ionic displacements are restrained by the short-ranged interactions with the nearest neighbors.^64^ For both PYR_14_ and TFSI, the extension of these regions varied significantly on going from SPE5 to SPE2, for example, increasing the Li content. A similar outcome was also observed in SPE4 and SPE3. The regions sampled by Li cations were, conversely, much less affected by the change in the Li content. As we shall show below, in most of the systems considered, a large fraction of Li cations essentially remained within the IL phase. Based on this outcome and our previous results, we conclude that Li/TFSI short-range coordination affects TFSI diffusion and, consequently, PYR_14_ diffusion. In systems with a high Li content, such as SPE2, PYR_14_ cations likely experience an enhanced caging effect because of the Li/TFSI coordination. This explains the observed decrease in PYR_14_ diffusion coefficients on going from SPE5 to SPE2.

An additional analysis was performed to evaluate the coordination between Li and PEO oxygens. Oxygen atoms in PEO have been shown to closely coordinate to Li ions in both PEO–Li salt binary^65,66^ and ternary systems.^20,21^ Inspection of the MD trajectories shows that a fraction of Li cations is effectively coordinated to PEO oxygens. For this reason, we calculated the average number of Li–O interactions within a 0.3 nm cutoff, corresponding to the extension of the first coordination shell. In Table 3, we report the average number of Li cations coordinated by at least one oxygen (*N*_Li_) and that of coordinating atoms (*N*_O_). *N*_Li_ was found to increase nonlinearly on going from SPE5 to SPE2. For the SPE2 system, 10 Li ions, corresponding to 16.7% of the total, were coordinated by about 2–3 PEO oxygens. On the opposite side, only one Li atom was coordinated by four–five oxygens in SPE5.

**Table 3:** Average Number of Coordinated Li, *N*_Li_, and Coordinating Oxygen, *N*_O_, Atoms Obtained from the MD Simulations at 19 °C^a^

sample	EO/Li/IL mole ratio	*N*_Li_	*N*_O_
SPE2	210:60:60	10.00 ± 0.04	2.65 ± 0.06
SPE3	210:40:80	3.0 ± 0.2	2.35 ± 0.13
SPE4	210:30:90	2	3.49 ± 0.02
SPE5	210:24:96	1	4.23 ± 0.55

a Standard deviations are reported in parentheses. The cutoff distance was set at 0.3 nm in all calculations.

This outcome is opposite to that found in other ternary systems, such as PEO/LiPF_6_/BMIMPF_6_,^67^ where the increase of IL content was found to increase the Li–PEO association because of the weakening of the lithium salt coordination.

It is worth noting that, in all systems investigated, the coordination between Li and PEO oxygens was very stable, as testified by the small fluctuations of *N*_O_ over time (see Figure S6). A further analysis revealed that both the coordinated and coordinating atoms were essentially the same during the timespan of the simulations. This outcome is consistent with that found in PEO–LiTFSI systems.^66^ Meanwhile, in analogy with another study on IL-free systems,^55^ we found that the Li ions in our systems were all coordinated by single PEO chains, regardless of the Li content. It should be noted that coordination to PEO oxygens was not exclusive. The Li atoms coordinated by PEO were also coordinated by TFSI anions, providing a possible explanation for the stability of ternary mixtures as opposed to IL–PEO binary ones.

As a last step in our investigation, we considered Li diffusion, which is key to the application of SPEs in batteries. Table 4 collects the average diffusion coefficients, *D*^sim^, calculated by means of eq 5, at *t* = 400 ns. The Li diffusion coefficient increases with the IL content, except for SPE4 which showed the maximum value of *D*^sim^, regardless of the choice of the correlation time (see Figure S7). We note that our trend is analogous to that experimentally observed by Joost^15^ et al. on analogous systems, albeit at the temperature of 50 °C. The increase in Li mobility with IL fraction is consistent with the results presented above (see Figure 11) and mainly related with Li/TFSI coordination. Increasing the IL content improves TFSI, PYR_14_, and Li mobility as well. The result obtained for SPE4 suggests a more complicated picture, probably involving a delicate balance between the composition and transport behavior. Finally, we note that our results for Li mobility agree with those obtained by Diddens^20,21^ and Heuer, despite the fact that these authors excluded the possibility that Li diffusion could develop within the IL phase. Based on this observation, we conclude that both media can be equally suitable for Li transport, with the preference for one or the other probably determined by the local mixture composition.

**Table 4:** Calculated *D*^sim^ Average Diffusion Coefficients at 19 °C for Li, Calculated Integrating *P*(*D*) Profiles via Eq 5, at *t* = 400 ns

sample	EO/Li/IL mole ratio	*D*^sim^ [10^–6^ mm^2^/s]
SPE2	210:60:60	0.00845
SPE3	210:40:80	0.0122
SPE4	210:30:90	0.0247
SPE5	210:24:96	0.0197

## 4 Conclusions

In this work, we have investigated the dynamics of the PYR_14_ and Li cations in PEO/LiTFSI/PYR_14_TFSI electrolyte blends by means of MRI techniques and MD simulations. In order to evaluate the effect related to the IL content, we considered samples with increasing PYR_14_TFSI mole ratio. The analysis of dMRI and *T*_2_-MRI images clearly evidenced the spatially heterogeneous distribution of diffusion coefficients and relaxation times of PYR_14_ within the polymer matrix, supporting the idea of constrained PYR_14_ for both diffusion and molecular tumbling. Increasing the IL content, we found the PYR_14_ average diffusion coefficients to increase systematically, although well below the value estimated for the neat IL. Similarly, the measured *T*_2_ as a function of the PYR_14_TFSI molar content showed gradually increasing *T*_2_ but nonetheless significantly smaller than that of the bulk IL. To the best of our knowledge, this is the first time that MRI techniques have been used to get a detailed, spatially resolved view of molecule-specific dynamics in SPE composite materials. The constrained translational and rotational dynamics of PYR_14_ in the membrane is consistent with some type of interaction with the polymeric backbone.

In order to better rationalize the experimental observations, we performed MD simulations on closely related systems. The trend in the calculated diffusion coefficients for PYR_14_ is consistent with that obtained from MRI data. The inspection of the diffusivity profiles revealed the absence of multiple transport regimes on a time scale, which is half a microsecond. In neat ILs, ion dynamics is often mediated by the formation of transient cages formed by nearest neighbor molecules. We have shown that a similar model also applies to ILs in SPE systems, where the presence of Li has a noticeable effect because of its small size.

As far as Li diffusion is concerned, we have shown that the major fraction of Li ions remains within the IL phase during the MD simulations. A significant fraction of Li ions was coordinated by PEO oxygen atoms only in the SPE2 system. A connection between the plasticizing effect of PYR_14_TFSI on the binary PEO–LiTFSI system and the increase in Li diffusion cannot be established on the basis of our MD simulations. Besides, our study suggests that the IL also may provide a suitable medium for Li diffusion, thus providing the IL with a functional role going beyond the mere additive for inhibiting the growth of the polymer crystalline domains.

## Supporting Information Available

Pictures of sealed capillaries containing NMR samples; MRI screenshot of the sample positioning along the instrumental *z*-axis showing the slice thickness; proton density-weighted MRI images and histograms; plots showing the time evolution of diffusion coefficients of hydrogen atoms during the MD simulations; time-resolved coordination of lithium ions to PEO oxygens; plot showing the time evolution of the average diffusion coefficients for Li ions; and MRI field homogeneity assessment figures (PDF)
