# Supplementary material for: Magnetic Resonance Imaging and Molecular Dynamics Characterization of Ionic Liquid in Poly(ethylene oxide)-Based Polymer Electrolytes
Source: ACS Appl Mater Interfaces. 2020 Apr 30;12(21):23800–11. doi: 10.1021/acsami.0c01890 (PMC8007074; doi:10.1021/acsami.0c01890)
Supplement: Supplementary file 1 — am0c01890_si_001.pdf [file am0c01890_si_001.pdf]

SUPPORTING INFORMATION

MAGNETIC RESONANCE IMAGING AND MOLECULAR DYNAMICS CHARACTERIZATION OF IONIC LIQUID IN PEO-BASED POLYMER ELECTROLYTES

Mosè Casalegno[a,], Franca Castiglione[a,*], Guido Raos[a], Giovanni Battista Appetecchi[b], Stefano Passerini[c], Andrea Mele[a], and Enzio Ragg[d].*

[a] Dipartimento di Chimica, Materiali e Ing. Chimica "G. Natta", Politecnico di Milano, 20131 Milano, Italy

[b]ENEA, Italian National Agency for New Technology, Energy and Sustainable Economic Development, Materials and Physicochemical Processes Technical Unit, Via Anguillarese 301, 00196 Rome, Italy

[c]Helmholtz-Institut Ulm (HIU), Karlsruher Institut fír Technologie (KIT), Ulm, Germany; Karlsruhe Institute of Technology, P.O. Box 3640, 76131 Karlsruhe, Germany

[d]Dipartimento di Scienze Molecolari Agroalimentari, Università di Milano, 2Milano, Italy

Materials and sample preparation.

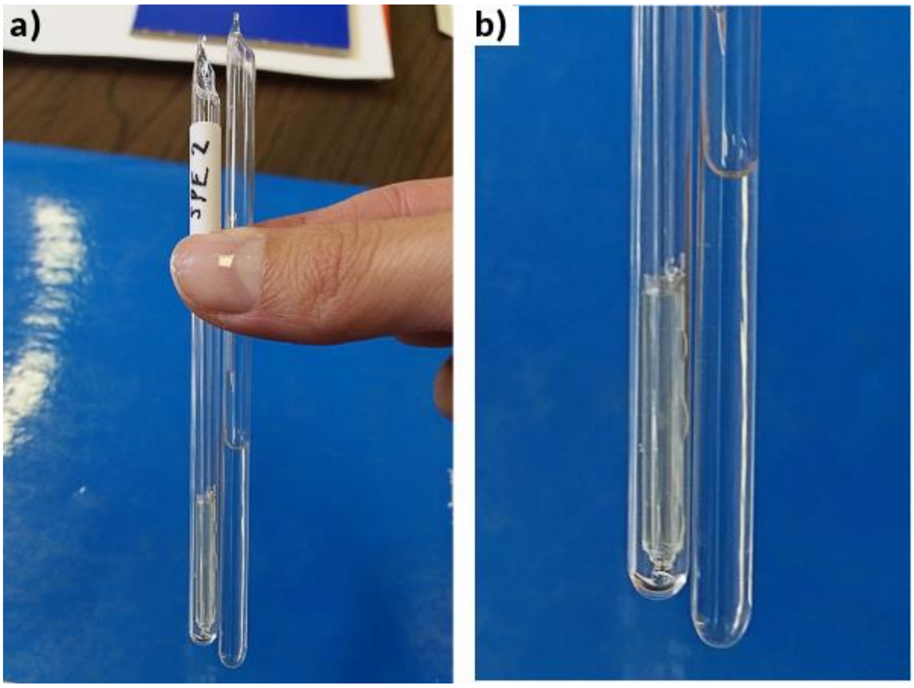

Figure S1. a) pictures of glass tubes containing PEO-LiTFSI-$PYR_{14}TFSI$ polymeric tape wrapped around a sealed internal capillary containing DMSO-d6 (left) and the $PYR_{14}TFSI$ ionic liquid with a sealed capillary containing DMSO-d6 (right). b) close-up views of the same glass tubes.

MRI characterization.

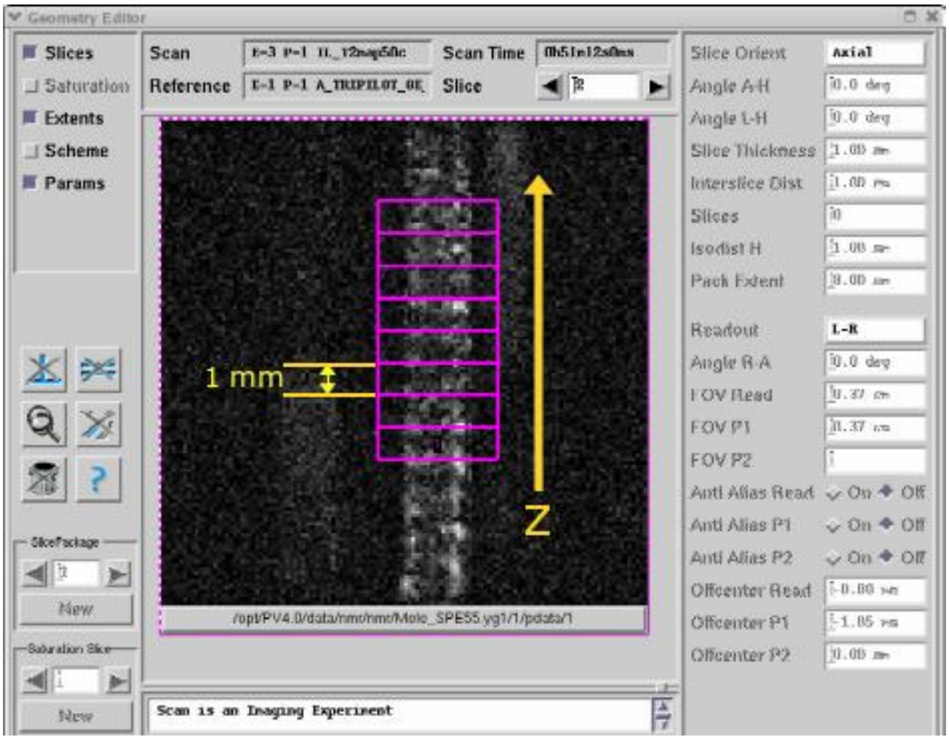

Figure S2. Screenshot showing the MRI sampling of the sample along the z-axis. A grid with 1 mm spacing has been added to better clarify the slice thickness used while collecting data (2 mm).

Proton density-weighted MRI images and histograms.

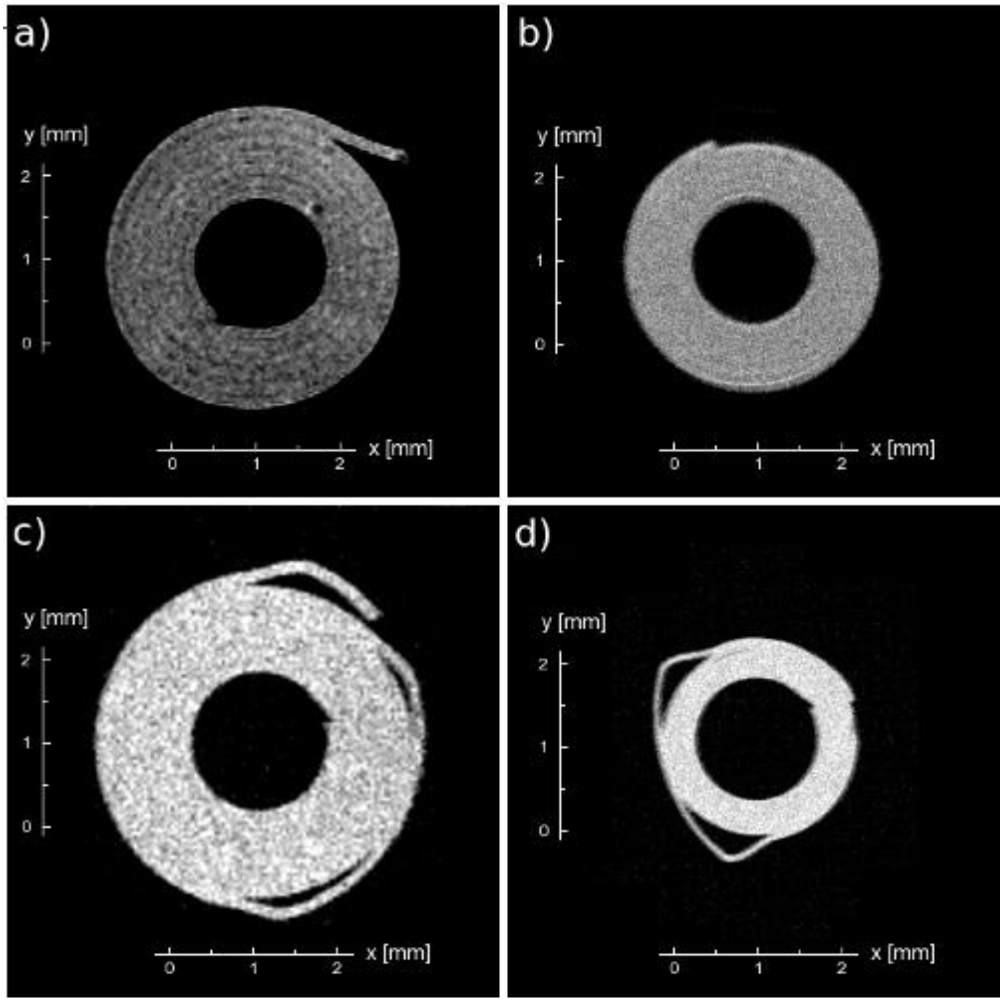

Figure S3. Proton density-weighted MRI images at 19 °C for the samples: a) SPE2, b) SPE3, c) SPE4, and d) SPE5.

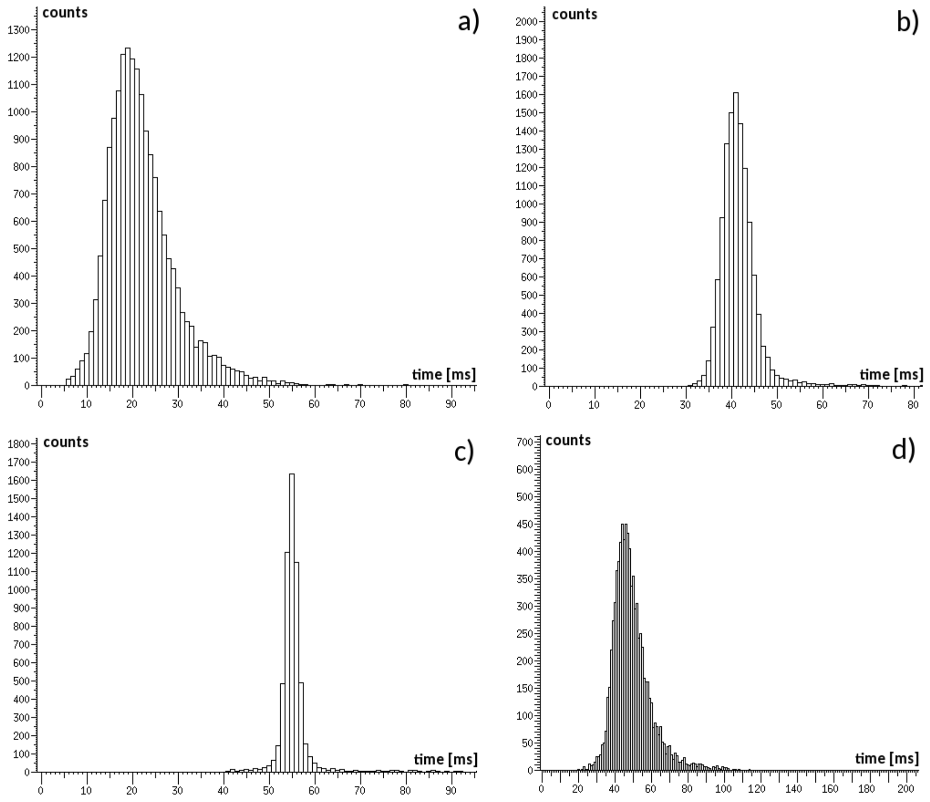

Figure S4. T_2 histograms at 19 °C for the samples: a) SPE2, b) SPE3, c) SPE4, and d) SPE5.

Time evolution of the average diffusion coefficients for hydrogen atoms of PYR_{14}.

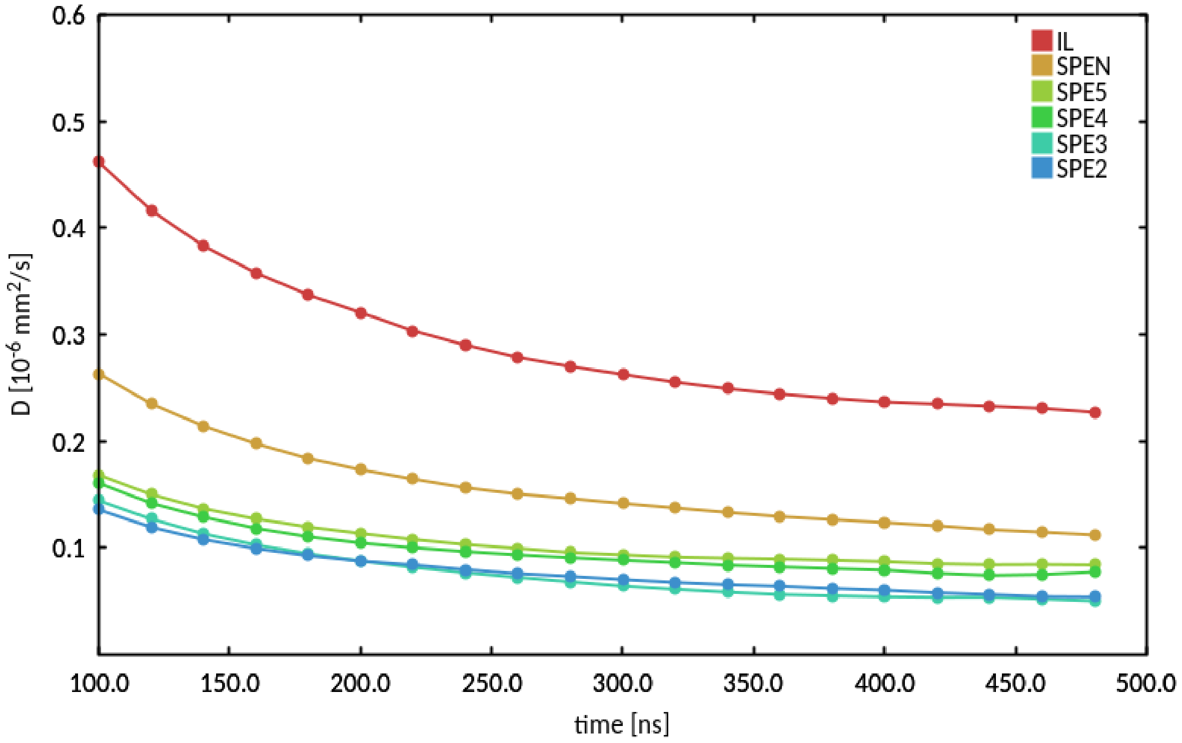

Figure S5. Plot of the average diffusion coefficients of hydrogen atoms (PYR_{14}) calculated by means of Eq. (5) for all the systems investigated at different correlation times. The values at t = 400 ns were as D^{sim} in Table 2.

Time-resolved coordination of Lithium ions to PEO oxygens.

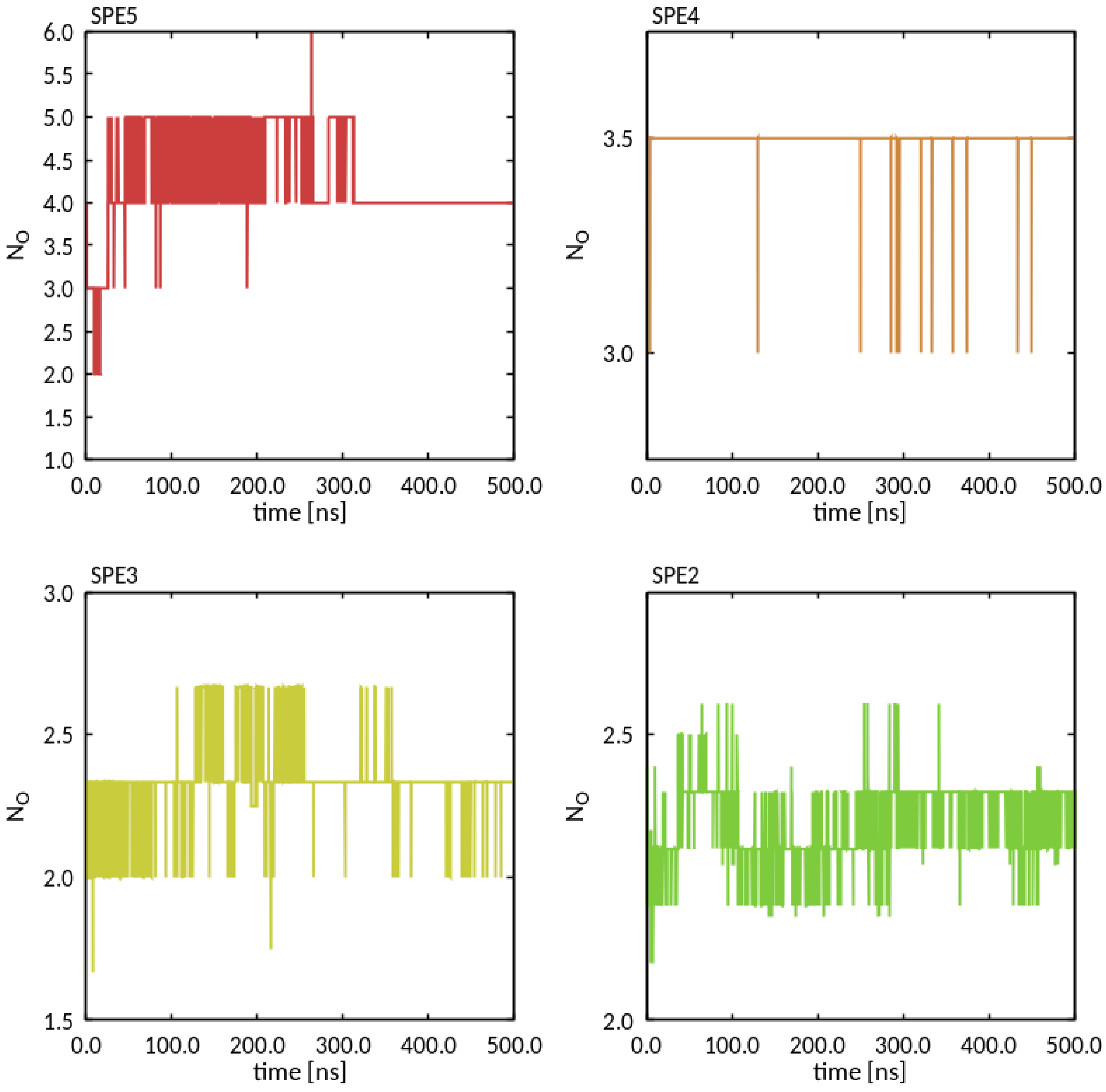

Figure S6. Time evolution of the number of coordinating oxygens per Lithium ion (N_O) for the ternary systems: SPE5, SPE4, SPE3, and SPE2.

Time evolution of the average diffusion coefficients for Li ions.

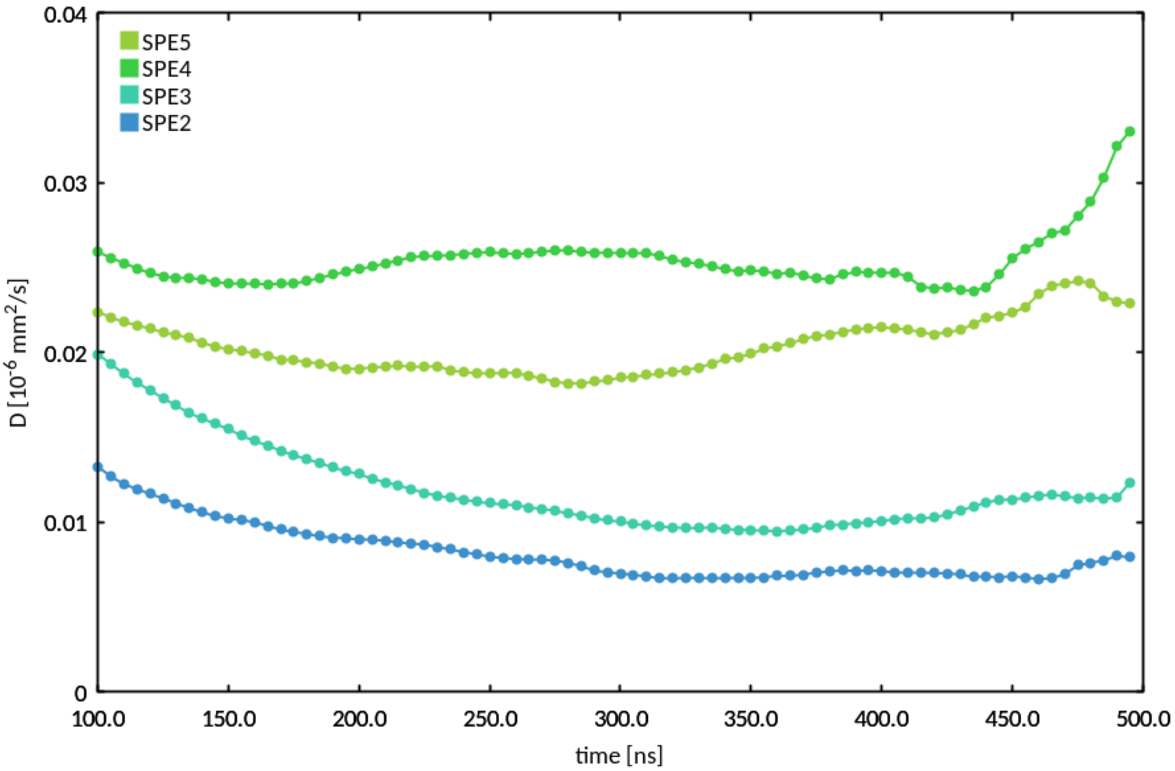

Figure S7. Plot of the average diffusion coefficients of Li calculated by means of Eq. (5) for all the systems investigated at different correlation times for the ternary systems: SPE5, SPE4, SPE3, and SPE2. The values at t = 400 ns were reported as D^{sim} in Table 4.

MRI field homogeneity assessment.

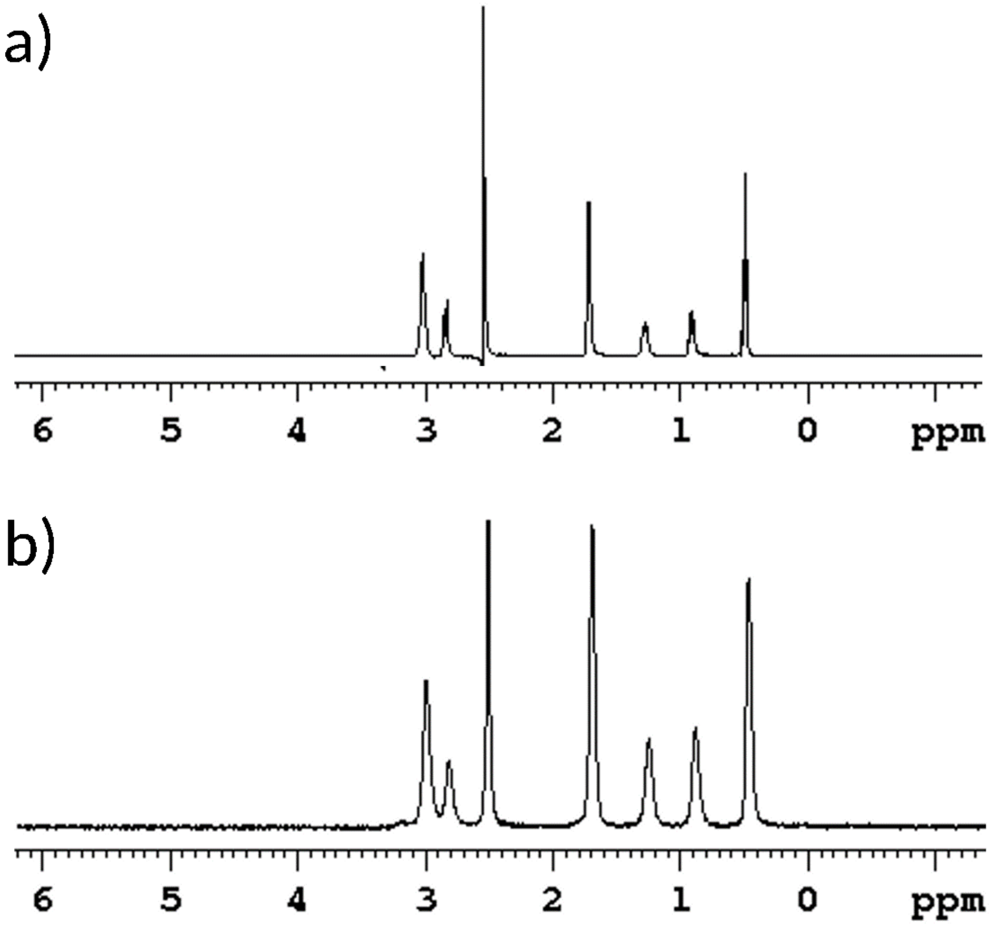

Figure S8. ^{1}H-NMR spectra acquired using a volume selective spectroscopy MRI protocol. a) IL (neat); b) SPE5 sample. Experimental conditions: MRI method: PRESS (Bruker Paravision library). T =294 K. Sample volume: 1 mm^3; echo time: 20 ms; number of scans: 64; Spectral width: 20 ppm; time domain: 16 K. Apodization: 0.5 Hz exponential multiplication. Both samples were sealed under vacuum in a standard 5 mm (O.D.) NMR tube to avoid water contamination as shown in Figure S1.

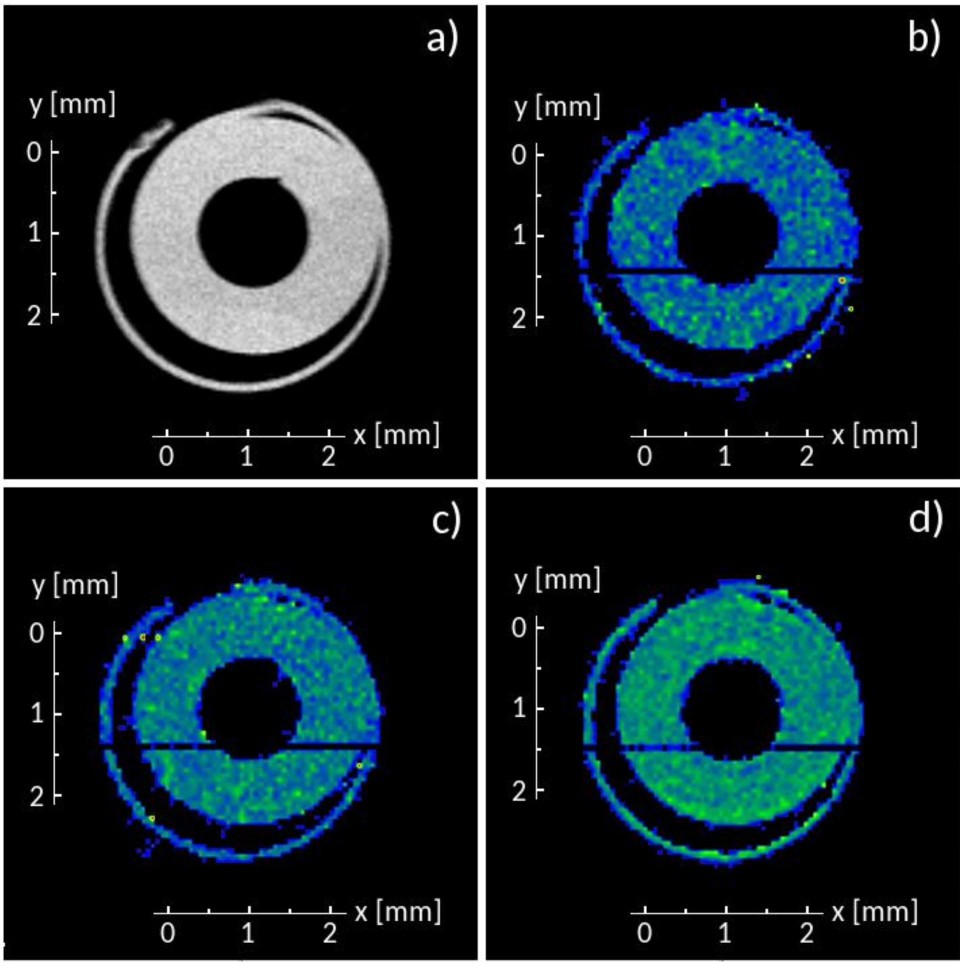

Figure S9. MRI Diffusion maps for SPE4 measured at different gradient separation values (Δ) for the evaluation of background gradients. Sample temperature set at T = 294 K. a) proton density map. b) Δ = 0.4 s; c) Δ = 0.3 s; d) Δ = 0.2 s. In all measurements the gradient duration (δ) was set at 5 ms. Diffusion values have been color coded in 256 color gradations from blue (0.35×10^{-6} mm^2/s) to green (3.90×10^{-6} mm^2/s) to red (7.25×10^{-6} mm^2/s).

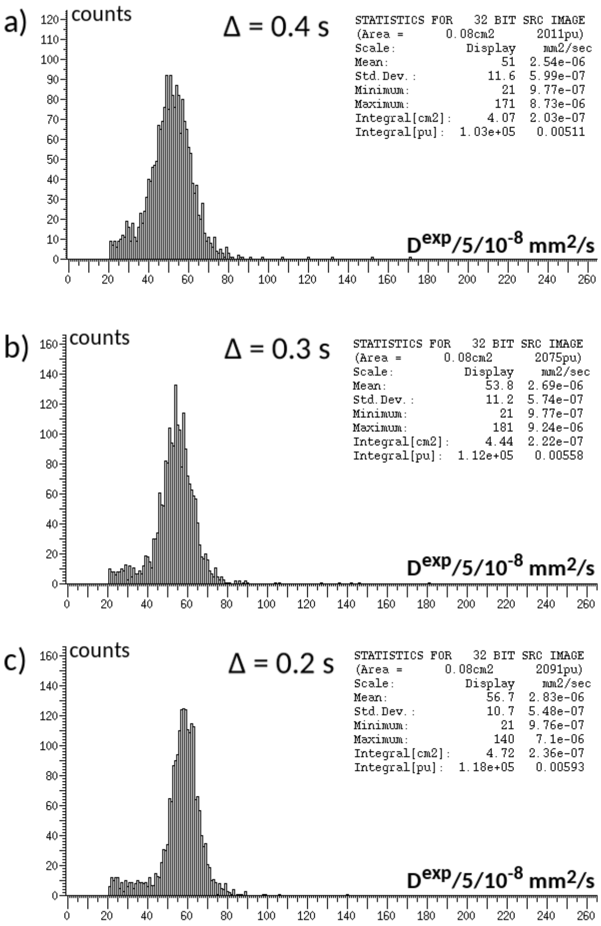

Figure S10. Diffusivity histograms for the SPE4 sample, corresponding to the DW images reported in Figures S9 (panel a to Fig. S9b, panel b to Fig. S9c, and panel c to Fig. S9d).

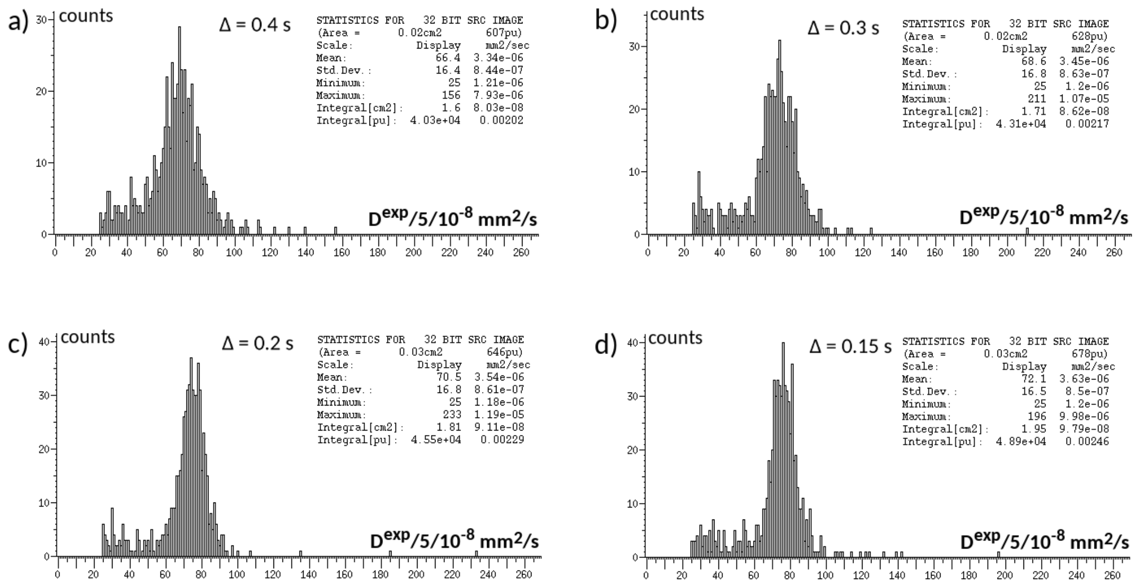

Figure S11. Diffusivity histograms of DW diffusion values for the SPE5 sample at different gradient separation values, (Δ, shown on top). T = 293 K. For Δ values between 0.4 s and 0.2 s the gradient duration (δ) was set at 5 ms; for Δ = 0.15 s, δ = 5.5 ms. The remaining acquisition parameters are as in Fig. S8.
